# The transcription factor RttA contributes to sterol regulation and azole resistance in *Aspergillus fumigatus*

**DOI:** 10.1128/mbio.01854-25

**Published:** 2025-09-12

**Authors:** Lukas Birštonas, Peter Hortschansky, Ingo Bauer, Ervin M. Alcanzo, Alexander Kühbacher, Birte Mertens, Christoph Müller, Axel A. Brakhage, Fabio Gsaller

**Affiliations:** 1 Institute of Molecular Biology, Biocenter, Medical University of Innsbruck 27280, Innsbruck, Austria; 2 Department of Molecular and Applied Microbiology, Leibniz Institute for Natural Product Research and Infection Biology (Leibniz-HKI) 28406, Jena, Germany; 3 Department of Pharmacy-Center for Drug Research, Ludwig-Maximilians-Universität München 9183, Munich, Germany; Universidade de Sao Paulo, Ribeirao Preto, Sao Paulo, Brazil

**Keywords:** *Aspergillus fumigatus*, transcription factor, ergosterol biosynthesis, azole resistance

## Abstract

Major mechanisms of azole resistance in *Aspergillus fumigatus* involve overexpression of the azole target protein encoded by *cyp51A*. The elevated expression originates from the duplication of an enhancer element in its promoter, serving as a binding platform for AtrR and SrbA, two central transcription factors that orchestrate the activation of ergosterol biosynthesis genes and azole resistance. Alongside AtrR and SrbA, several other transcription factors were shown to be crucial to mediate azole tolerance. Here, we unveil RttA as a further protein involved in the regulation of ergosterol biosynthesis and azole resistance in *A. fumigatus*. Because the gene was wrongly annotated, its encoded protein remained a protein with unclear function. Based on mapped RNA-seq reads, the coding sequence was corrected, yielding a DNA-binding protein comprising a Zn_2_Cys_6_ binuclear zinc cluster. Domain analysis and structural comparisons implied similarity between RttA and *Neurospora crassa* NcSR and yeast Upc2, both involved in sterol regulation and azole resistance. Through deletion and overexpression of *rttA*, we confirm its role in azole resistance. Transcriptional profiling of *atrR*, *srbA*, and *rttA* deletion mutants revealed that *rttA* expression depends on both AtrR and SrbA. In addition, our analyses uncovered a positive regulatory role for RttA in the expression of efflux pump-encoding genes and sterol homeostasis through activation of *erg6* expression. In agreement, the lack of *rttA* led to a substantial accumulation of the Erg6 substrate lanosterol. Collectively, this work elucidates RttA as a transcription factor in the clinically important fungal pathogen *A. fumigatus* involved in the regulation of ergosterol biosynthesis and azole tolerance.

**IMPORTANCE:**

Azole antifungals are frontline treatments against *Aspergillus fumigatus*, a major cause of life-threatening fungal infections. Resistance to azoles is a growing concern, often linked to increased expression of *cyp51A*, which encodes the azole target enzyme. This upregulation depends on the transcription factors AtrR and SrbA, key activators of ergosterol biosynthesis genes. Here, we identify and characterize a previously misannotated gene, *rttA*, encoding a Zn₂Cys₆ transcription factor structurally related to Upc2 and NcSR, sterol regulators in yeast and *Neurospora crassa*. Functional analyses, including gene deletion, overexpression, and transcriptomics, show that RttA promotes azole resistance and regulates sterol homeostasis by activating *erg6*, encoding sterol C24-methyltransferase. Loss of *rttA* leads to lanosterol accumulation, indicating disrupted ergosterol biosynthesis. Moreover, *rttA* expression depends on both AtrR and SrbA, placing RttA within their regulatory network. Our findings offer new insight into sterol regulation and antifungal resistance in *A. fumigatus*, highlighting RttA as a novel regulator.

## INTRODUCTION

New estimates suggest that the previous figure of 1.5–2.0 million deaths per year due to fungal diseases was heavily underestimated. The annual death toll is now predicted to be as high as 3.8 million, with *Aspergillus* infections alone being associated with more than 2 million deaths (1–3). Although several *Aspergillus* species can cause human disease, *Aspergillus fumigatus* is the major cause of human aspergillosis (4–6). Its ubiquitous nature, its ability to adapt to hostile environments, including the host, and possession of a large number of virulence factors are important features that have made this species the most important human mold pathogen worldwide (7, 8). Current therapeutics for the treatment of aspergillosis are limited to only three main classes of antifungal agents, including the triazoles, which are typically first-line treatment (9, 10). Azole antifungals inhibit the production of ergosterol, a crucial compound of the fungal cell membrane that contributes to its permeability and integrity (11). The initial building block of ergosterol biosynthesis represents acetyl-CoA, which is converted through several enzymatic steps into farnesyl-pyrophosphate, an intermediate that is required as substrate for several other downstream products such as heme, dolichol, and ubiquinone (12, 13). Specific ergosterol biosynthesis begins with the condensation of two farnesyl-pyrophosphate units into squalene, catalyzed by squalene synthase Erg9. Squalene is then converted by squalene monooxygenase Erg1 into 2,3-oxidosqualene, which is subsequently transformed into lanosterol by lanosterol synthase Erg7 (12, 13). While in yeast lanosterol is the main substrate of sterol C14-demethylase (named Erg11 in yeast), in *A. fumigatus* lanosterol is first converted by sterol C24-methyltransferase Erg6 into eburicol, the substrate of the sterol C14-demethylase (named Cyp51 in *A. fumigatus*) in this species (12, 14). Inhibition of Cyp51 leads to ergosterol depletion as well as increased levels of eburicol (14–16). Accumulation of the latter favors an increased production of toxic sterols (14, 17, 18). The *A. fumigatus* genome encodes two Cyp51 isoenzymes, Cyp51A and Cyp51B, that catalyze eburicol C14-demethylation. Resistance, however, is predominantly associated with mutations in the *cyp51A* gene (19–21).

In *A. fumigatus,* transcriptional regulatory mechanisms play a critical role in azole resistance. In fact, most prevalent mechanisms of resistance, termed TR34/L98H and TR46/Y121F/T289A, involve overexpression of *cyp51A* (22, 23). Both TR34 and TR46 are tandem repeat (TR) mutations within the *cyp51A* promoter that contain a duplicated transcriptional enhancer element (24, 25). The respective DNA stretch harbors binding sites for the sterol regulatory element-binding protein (SREBP) SrbA and the ATP-binding cassette (ABC)-transporter-regulating transcription factor AtrR. Their contribution to azole tolerance has been intensively investigated over the past years; thereby, they have been identified as key transcription factors required for the activation of multiple ergosterol biosynthesis genes, including *cyp51A* (26–30). Although both regulators have multiple overlapping target genes, a unique target gene of AtrR is *cdr1B*, a major efflux pump that contributes to azole antifungal resistance (29, 31). Based on their critical role in *A. fumigatus* azole resistance, both proteins or their associated network were suggested as attractive targets for combination therapy with azole antifungals, which was further corroborated as inactivation of either SrbA or AtrR rendered *A. fumigatus* isolates carrying *cyp51A* TR alleles azole susceptible (32, 33). Several years ago, screening of the first *A. fumigatus* transcription factor knock-out library against azoles led to the discovery of a set of several further transcription factors that were crucial determinants of azole resistance (34). The outcomes of this study highlighted that a more complex regulatory network defines the naturally occurring tolerance of *A. fumigatus* to azole antifungals. Nevertheless, the severe azole susceptibility phenotypes of ∆*srbA* and ∆*atrR* highlighted the respective proteins as critical transcription factors contributing to azole tolerance in wild type (34).

More than a decade ago, the gene *AFUA_7G04740* (*AFUB_090280*) encoding a protein with at that time unknown function was found among a set of 87 genes to be significantly downregulated in ∆*srbA* during hypoxia (28). Several years later, through a ChIP-seq approach aiming to identify genes under direct control of the SrbA protein during hypoxia, high enrichment of SrbA in the 5′ upstream region of the respective gene was detected, suggesting SrbA as its direct regulator (35). Only recently, this gene has been described in relation to tolerance to the azole pesticide tebuconazole (36). In the respective study, the gene was termed *rttA*, derived from responsible for tebuconazole tolerance. Further implying a role of this gene in response to azole exposure, *rttA* transcript levels were elevated in *A. fumigatus* during itraconazole exposure (36) and voriconazole persister cells, together with several ergosterol biosynthesis genes and the azole efflux pump *cdr1B* (37). Its homology to Cys6 zinc finger proteins in other *Aspergillus* species has been discussed, but due to the absence of the respective DNA-binding domain, its potential role as a transcription factor could not be further elucidated (36). The currently publicly available protein sequences of RttA in the isolates A1163 (GenBank: EDP48313.1) and Af293 (GenBank: EAL86998.1) are 309 amino acids in size, encoded by a gene that contains two exons and one intron. Exploiting mapped RNA-seq read data, in this work, we corrected its coding sequence, which leads to a deduced protein with an extended N-terminus comprising a Zn_2_Cys_6_ binuclear zinc cluster. This finding strongly suggested a role for this protein as a DNA-binding transcription factor. Structural similarity predictions implied homology to *Neurospora crassa* NcSR (38) as well as Upc2 homologs in *Saccharomyces cerevisiae* and pathogenic yeast species (39–47). Disruption and overexpression of *rttA* confirmed its apparent role in azole resistance. With suspected function in the regulation of ergosterol biosynthesis, we found RttA to specifically drive *erg6* expression. In accordance, the Erg6 substrate lanosterol was severely increased in an *rttA* deletion mutant.

## RESULTS

### Re-annotation of *rttA* leads to a deduced fungal-type Zn_2_Cys_6_ binuclear zinc cluster containing a transcription factor with structural similarities to yeast Upc2 and *Neurospora crassa* NcSR

**Fig 1:**
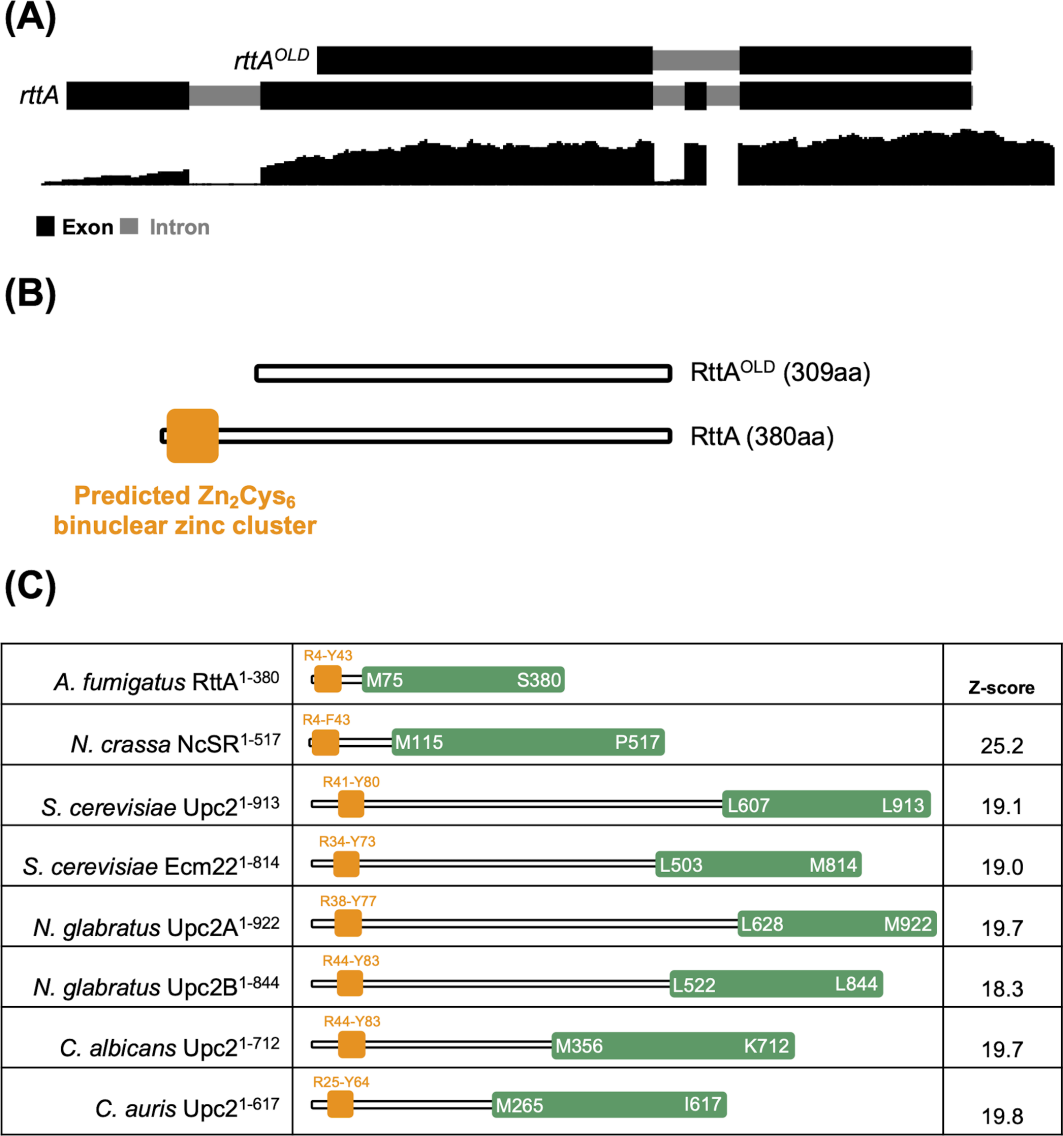
Re-annotation of *rttA* reveals a protein with a Zn_2_Cys_6_ binuclear zinc cluster at its N-terminus. (**A**) Schematic overview of the previously misannotated *rttA* (*rttA^OLD^*) and corrected annotation of the gene (*rttA*). Newly predicted *rttA* contains four instead of two exons, three instead of one intron, as well as an extension at the 5′ upstream region of the coding sequence. (**B**) The corresponding re-annotated protein contains an N-terminal Zn_2_Cys_6_ DNA-binding domain. (**C**) MUSCLE-based multiple sequence alignment of RttA, NcSR, and yeast Upc2 homologs was performed to predict the peptide regions of each protein overlapping the Zn_2_Cys_6_ binuclear cluster (orange) and the C-terminus containing the potential ligand binding domains (green). Z-scores comparing C-terminal RttA^75–380^ with the respective regions of Upc2 homologs and NcSR are displayed.

Most likely due to a misleading annotation of the gene, a clear functional categorization of *rttA* (AFUB_090280/AFUA_7G04740) could not be performed up to today. Comparing the currently annotated gene feature with RNA-seq read data generated in this work (Fig. 1A), it became apparent that the coding sequence was wrongly predicted. Using the mapped read data, we manually corrected the coding sequence, yielding a coding sequence translated into a protein (RttA) with an extended N-terminus of 60 amino acids and an additional 11 amino acids within the protein (Fig. 1B and Fig. S1). InterPro-based domain searches (48) using the new protein sequence suggested a Gal4-type Zn_2_Cys_6_ binuclear zinc cluster (InterPro entry IPR001138) at its N-terminus and a relation of RttA, like *S. cerevisiae* Upc2 and its paralog Ecm22, to the sterol uptake control protein family (InterPro entry IPR053157). Upc2 homologs are regulatory proteins that have received particular attention in pathogenic yeasts as key transcription factors involved in the regulation of ergosterol biosynthesis and azole antifungal resistance. These included *Nakaseomyces glabratus* (previously named *Candida glabrata*) Upc2A and Upc2B, *Candida albicans* Upc2, as well as *Candida auris* Upc2 (39–47). Further supporting a potential correlation of RttA and Upc2, BLAST analysis (49) against proteins of the baker’s yeast *S. cerevisiae* (taxid:559292) using the newly annotated sequence returned both Upc2 and Ecm22 as the best hits.

Interestingly, recent work suggested structural similarities between *S. cerevisiae* Upc2 and NcSR, a transcription factor in the filamentous model fungus *Neurospora crassa* that contributes to sterol regulation and azole tolerance (38). Like *A. fumigatus* SrbA and AtrR, *N. crassa* possesses SREBP (SAH-2) and AtrR homologs that participate, together with NcSR, in the regulation of sterol biosynthesis in this species (38). Thus, we speculated that NcSR could have similar functions to RttA. To identify conserved regions in RttA with potential similarities to NcSR as well as yeast Upc2 homologs, we first performed a MUSCLE (MUltiple Sequence Comparison by Log-Expectation)-based (50) sequence alignment with the abovementioned proteins (Fig. S1). The alignment highlighted protein sections at the N-terminus (amino acids 4–43: RttA^4–43^) overlapping the predicted Zn_2_Cys_6_ binuclear cluster DNA binding domain and the C-terminus (amino acids 75–380: RttA^75–380^), overlapping the characterized ligand binding domains (LBDs) of *S. cerevisiae* Upc2 and *N. glabratus* Upc2A. The respective LBDs have been elucidated in previous work as ergosterol-binding domains that determine Upc2 regulatory activity through its subcellular localization, depending on ergosterol availability (51, 52). Utilizing the multiple sequence alignment, we predicted the C-terminus containing LBD of each protein (Fig. 1C) and used AlphaFold3 (https://alphafoldserver.com) to model protein structures of the respective protein section for structural comparisons. To assess structural similarities, the generated structure model of RttA^75–380^ was pairwise compared to the models of the other C-terminal protein sections using the DALI (Distance-matrix ALIgnment) protein structure comparison server (http://ekhidna2.biocenter.helsinki.fi/dali/) (53). The retrieved Z-scores for C-terminal Upc2 homologs were in a range between 18.3 and 19.8 and 25.2 for NcSR (Fig. 1C). Z-scores of 8–20 indicate that the proteins are likely homologous, a Z-score >20 suggests homologous functions of proteins (54).

### Deletion of *rttA* increases azole susceptibility, and its overexpression elevates azole resistance

**Fig 2:**
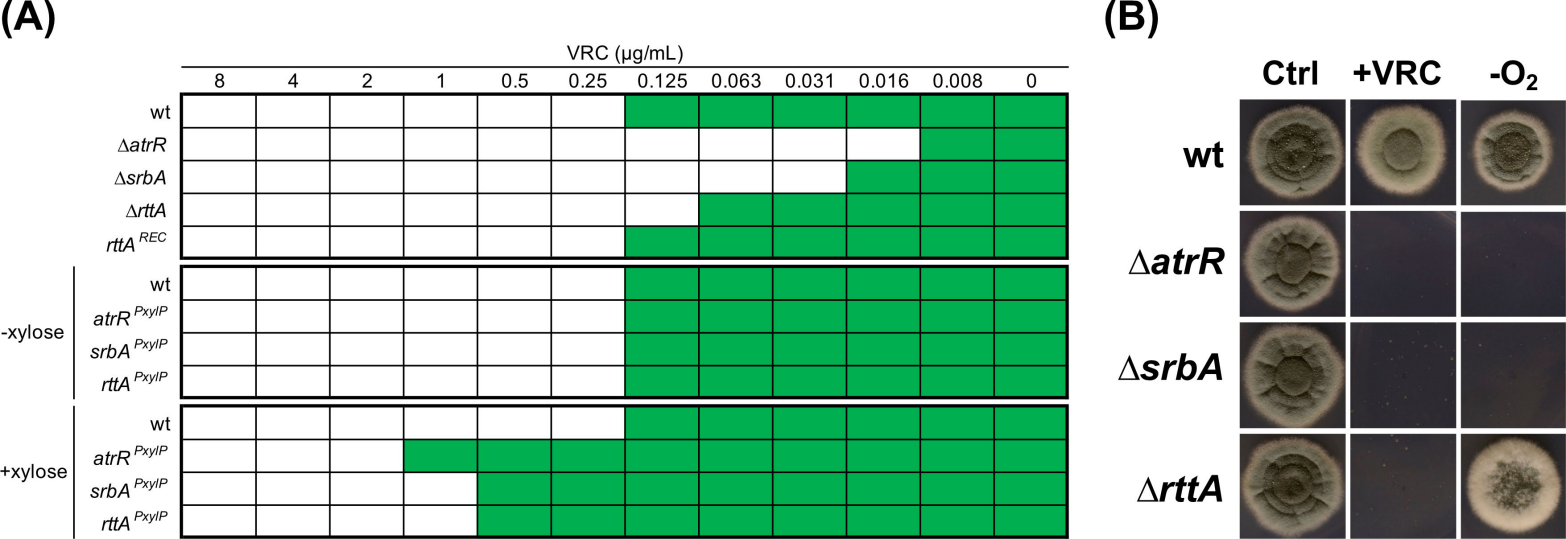
RttA is crucial for azole resistance but not hypoxia adaptation. (**A**) Compared to ∆*atrR* and ∆*srbA*, deletion of *rttA* results in a comparably low decrease in the voriconazole (VRC) MIC levels. Overexpression (+xylose) of *atrR*, *srbA,* and *rttA* increases VRC MICs several fold. MICs were determined following the procedure according to EUCAST (57). Growth was visually detected (green). (**B**) In contrast to ∆*atrR* and ∆*srbA*, growth of ∆*rttA* is barely affected during hypoxia (−O_2_). Strains were grown for 48 hours on solid *Aspergillus* minimal medium (AMM) at 37°C. +VRC, 0.15 µg/mL voriconazole.

Initially, a connection of *rttA* with azole tolerance was proposed as a non-synonymous mutation (A83T in RttA^OLD^; A143T located in the LBD of RttA) in the gene led to a slight decrease in azole susceptibility (36). To further unravel its significance in azole resistance, we generated an *rttA* null mutant (∆*rttA*) by deleting its coding sequence in wild type and compared its voriconazole susceptibility to deletion mutants of *srbA* (∆*srbA*) and *atrR* (∆*atrR*). In addition, to investigate effects resulting from elevated expression of these genes, we added additional *PxylP*-inducible (55) gene copies at the defined marker locus *fcyB* (56) into wild type (strains *rttA^PxylP^*, *atrR^PxylP^*, and *srbA^PxylP^*). Similar to what has been observed in preceding work (26, 28, 29, 32), disruption of *atrR* and *srbA* led to azole hyper-susceptibility with 8- to 16-fold lowered minimum inhibitory concentration (MIC) levels when compared to wild type (Fig. 2A). In agreement with previous findings that suggested only a moderate contribution of *rttA* to azole tolerance (36), deletion of the gene lowered the MIC only 2-fold. The reconstituted strain *rttA^REC^*, which was generated by integrating a functional *rttA* gene copy at the *rttA* deletion locus, showed wild-type-like azole susceptibility. Overexpression of *rttA* increased the MIC 8-fold, similar to that observed during *srbA* induction. With an MIC change of 16-fold, induction of *atrR* resulted in the highest degree of resistance. As a direct target of SrbA and hence the potential role in hypoxia adaptation, we further tested the growth of ∆*rttA* during oxygen depletion (Fig. 2B). In contrast to ∆*atrR* and ∆*srbA,* which are unable to grow during hypoxia, only a slight growth difference could be observed for ∆*rttA* during oxygen depletion when compared to wild type.

### RttA exerts a positive regulatory function on *erg6*, and the expression of its encoding gene depends on functional SrbA and AtrR

**Fig 3:**
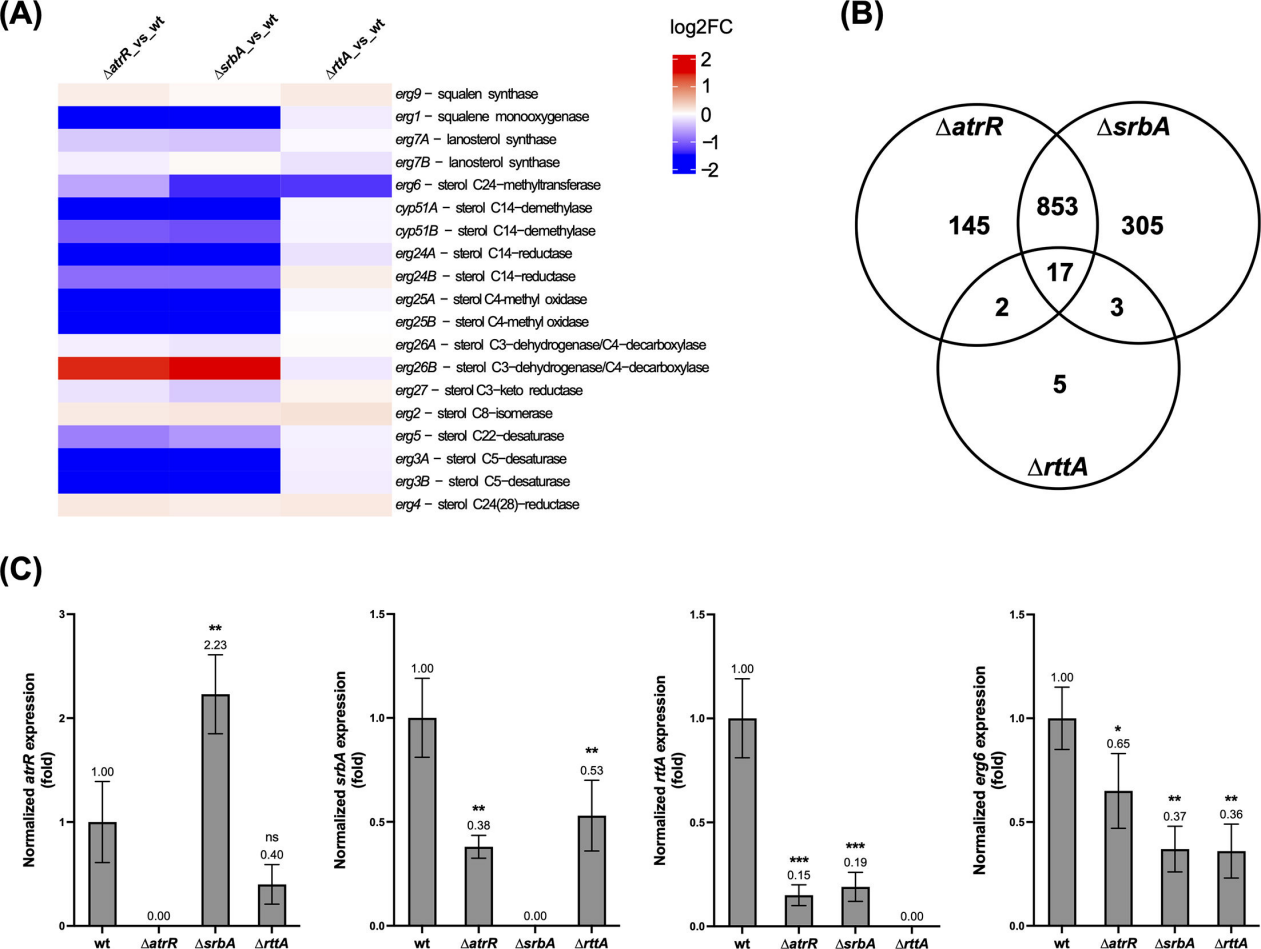
*rttA* expression depends on functional AtrR and SrbA, and its disruption results in decreased *erg6* transcript levels. (**A**) Heatmap illustrating differentially expressed genes of the ergosterol biosynthesis pathway in ∆*atrR*, ∆*srbA,* and ∆*rttA* compared to wild type (wt). (**B**) Venn diagram displaying the number of unique and commonly deregulated (>2-fold) genes in the transcription factor mutants. (**C**) Fold changes in expression compared to wild type (wt) were determined by RT-qPCR. For expression analysis, strains were grown in AMM for 18 hours at 37°C. Results represent the mean of biological triplicates normalized to wild type (wt), and error bars illustrate the standard deviation. *P*-values (pval) were calculated by one-way ANOVA. ***P < 0.0005, **P < 0.005, *P < 0.05, not significant (ns) *P* > 0.05.

In *A. fumigatus,* SrbA and AtrR are crucial for the activation of multiple ergosterol biosynthesis genes (28–30, 58). To compare potential overlapping regulatory functions with AtrR, SrbA, and RttA, particularly in the regulation of this pathway, transcriptional profiles of wt, ∆*atrR*, ∆*srbA,* and ∆*rttA* were generated. In contrast to ∆*atrR* and ∆*srbA*, displaying 1178 (815 down, 363 up) and 1017 (728 down, 289 up) genes significantly differentially expressed (>2 fold, adjusted *P*-value < 0.05), respectively, only the expression of a comparably small set comprising 27 (24 down, 3 up) genes was affected in ∆*rttA* (Table 1 and Table S2). This set included *AFUB_030790 (AFUA_2G15130*) encoding the ABC drug transporter AbcA (59) as well as its clustered gene *AFUB_030800* (*AFUA_2G15140*) encoding a putative MFS drug transporter. While *atrR* and *srbA* were not included in this set of deregulated genes in ∆*rttA*, we found *rttA* transcript levels several-fold decreased in ∆*atrR* and ∆*srbA*. The respective outcome was validated by RT-qPCR (Fig. 3C). In agreement with previous work (29, 30, 58), expression levels of numerous genes of the ergosterol-specific biosynthesis pathway were decreased in ∆*atrR* and ∆*srbA*. Only one ergosterol biosynthetic gene showed significant differential expression in ∆*rttA*, namely *erg6* encoding sterol C24-methyltransferase (Table 1 and Fig. 3A). Matching recent work (29), *atrR* expression was increased in ∆*srbA* and *srbA* expression was decreased in ∆*atrR* (Fig. 3C).

**TABLE 1:** Set of genes significantly deregulated >2-fold in ∆*rttA* compared to wild type^*a*^

Gene ID	Predicted gene function	log2 fold change ∆*rttA* vs wt	Adjusted *P*-value
AFUB_090280	Conserved hypothetical protein (*rttA*)	−9.89	1.12E-13
AFUB_033190	Cyanide hydratase/nitrilase	−3.02	1.23E-81
AFUB_030790	ABC multidrug transporter	−2.45	1.81E-144
AFUB_081430	Conserved hypothetical protein	−2.15	5.61E-04
AFUB_090430	Sterol glucosyltransferase	−2.05	1.47E-31
AFUB_018480	Short-chain dehydrogenase/oxidoreductase	−2.04	2.55E-25
AFUB_030800	MFS drug transporter	−1.70	1.43E-33
AFUB_100810	Conserved hypothetical protein	−1.35	8.36E-04
AFUB_099400	Sterol C24-methyltransferase	−1.30	4.66E-56
AFUB_085430	MFS sugar transporter	−1.30	2.32E-04
AFUB_001000	Conserved hypothetical protein	−1.24	5.51E-07
AFUB_092270	Flavin-containing monooxygenase	−1.21	6.27E-04
AFUB_097090	IgE-binding protein	−1.19	1.64E-05
AFUB_086160	Methionine aminopeptidase, type II	−1.16	4.24E-06
AFUB_044950	Conserved hypothetical protein	−1.15	1.48E-02
AFUB_044030	Cytochrome P450 monooxygenase	−1.13	2.54E-04
AFUB_046570	Porphyromonas-type peptidyl-arginine deiminase superfamily	−1.09	2.99E-23
AFUB_009550	Integral membrane protein	−1.09	1.33E-02
AFUB_090110	Na/K ATPase alpha 1 subunit	−1.08	1.87E-08
AFUB_060680	bZIP transcription factor (Atf21)	−1.07	1.17E-02
AFUB_062410	Fucose-specific lectin FleA	−1.06	4.29E-02
AFUB_073220	Purine-cytosine permease	−1.06	1.34E-04
AFUB_084640	Extracellular endo-polygalacturonase	−1.03	2.66E-03
AFUB_034300	Hypothetical protein	−1.00	3.89E-02
AFUB_071030	Metalloreductase	1.33	2.45E-05
AFUB_092700	RING finger protein	1.43	6.62E-04
AFUB_090300	Gamma-glutamyltranspeptidase	2.68	2.16E-160

^*a*^ For expression analysis, strains were grown in AMM for 18 hours at 37°C. The data represent log2 fold changes derived from DESeq2 analysis of biological triplicates. Statistical significance was assessed using the Wald test for each gene and corrected for multiple testing using the Benjamini-Hochberg procedure.

**Fig 4:**
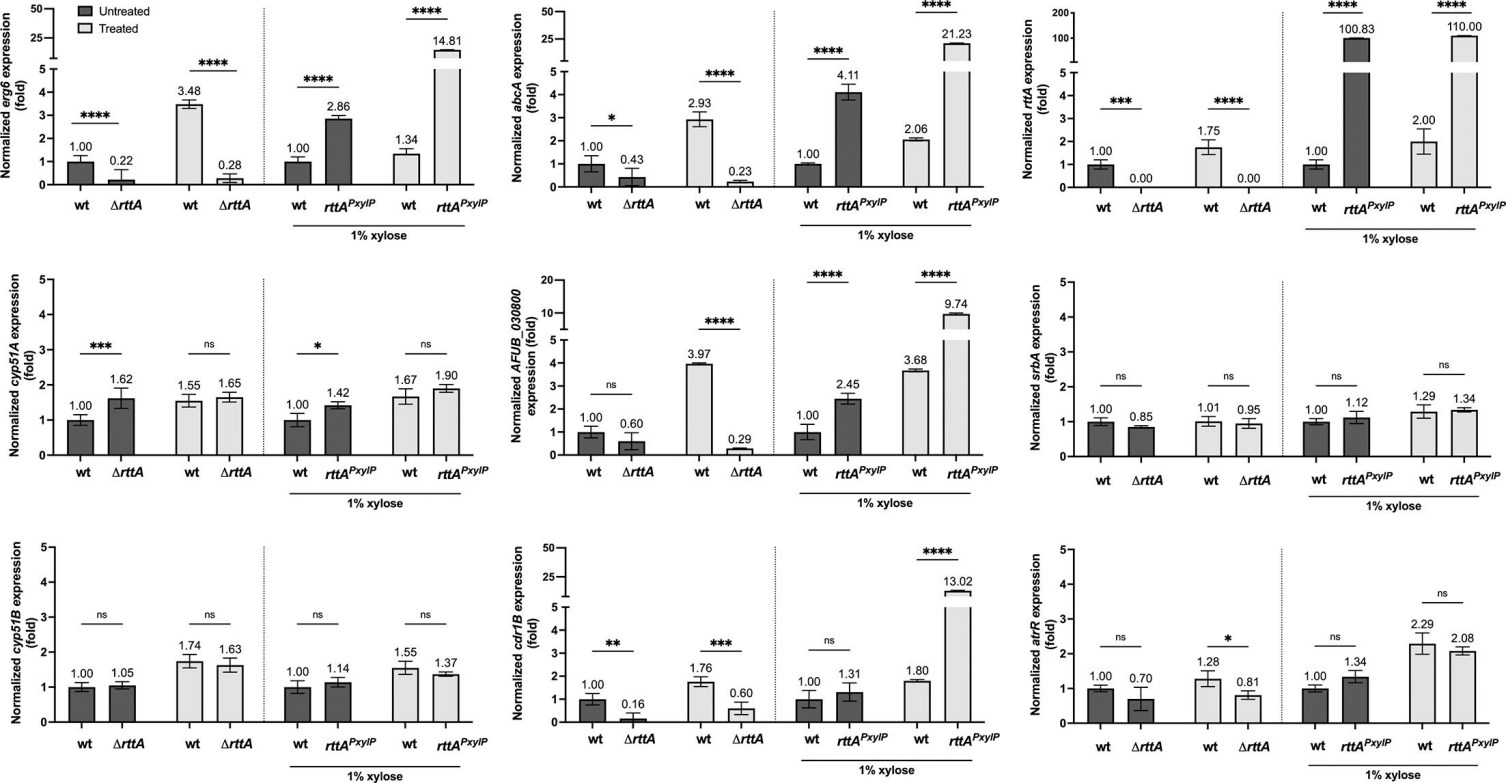
*erg6* but not *cyp51A* or *cyp51B* expression is severely affected in *rttA* mutants, particularly during azole exposure. RT-qPCR-based expression analysis of *erg6*, *cyp51A*, *cyp51B*, *abcA*, *AFUB_030800*, *cdr1B*, *rttA*, *srbA,* and *atrR* in ∆*rttA* and *rttA^PxylP^*. After pre-growth of strains in AMM for 17 hours at 37°C, 0.5 µg/mL voriconazole (Treated) was supplemented to the medium for a duration of 1 h. The respective amount of the solvent (DMSO, Untreated) was used in the controls. To induce *rttA* in *rttA^PxylP^*, 1% xylose was added to the medium. Results represent the mean of biological triplicates normalized to wild type (wt), and error bars illustrate the standard deviation. *P*-values (pval) were calculated by two-way ordinary ANOVA. ****P < 0.0001, ***P < 0.0005, **P < 0.005, *P < 0.05, not significant (ns) *P* > 0.05.

*N. crassa* NcSR was found to be crucial for activation of *erg6* as well as its *cyp51* ortholog (*N. crassa erg11*) (38). However, in this species, differential expression of both genes could only be observed during exposure of the mutant to azoles (38). Based on the idea that azole treatment and consequent depletion or accumulation of specific sterols might regulate RttA activity in *A. fumigatus*, we analyzed expression of *erg6* as well as *cyp51A* and *cyp51B* paralogs during voriconazole stress in wild type and ∆*rttA* (Fig. 4). To study potential positive regulatory effects on *erg6* transcript levels during *rttA* overexpression, we included the inducible *rttA* mutant *rttA^PxylP^* for expression analysis. In line with the transcriptional profile of wild type and ∆*rttA*, *erg6* transcript levels were decreased (4.5-fold) in the mutant grown in the absence of voriconazole. In contrast to wild type, *erg6* levels barely increased due to voriconazole exposure in ∆*rttA*, leading to an even bigger difference in its expression between wild type and mutant during azole stress (12.4-fold). In further agreement with a positive regulatory function of RttA on *erg6* expression, *rttA* overexpression elevated *erg6* expression (2.9-fold), and the effect was even more pronounced during voriconazole treatment (11.1-fold). With the exception of a slight increase in *cyp51A* expression in ∆*rttA* without azole stress, transcript levels of *cyp51A* as well as *cyp51B* were barely affected (<2-fold during all conditions tested). Similar to what has been observed previously (36), voriconazole exposure led to a moderate increase in *rttA* transcript levels in wild type.

In addition to *erg6* and the *cyp51* paralogs, we monitored transcript levels of *srbA*, *atrR*, *abcA*, *AFUB_030800* as well as *cdr1B* (Fig. 4). Expression of *srbA* and *atrR* was barely affected in both ∆*rttA* and *rttA^PxylP^. atrR* showed slightly decreased transcript levels in ∆*rttA* only during azole exposure. *abcA* and *AFUB_030800* showed a similar regulation pattern to that observed for *erg6. rttA* induction in *rttA^PxylP^* caused upregulation of *cdr1B*, particularly in the presence of voriconazole.

### Lack of RttA leads to high accumulation of the Erg6 substrate lanosterol and depletion of ergosterol during azole treatment

**Fig 5:**
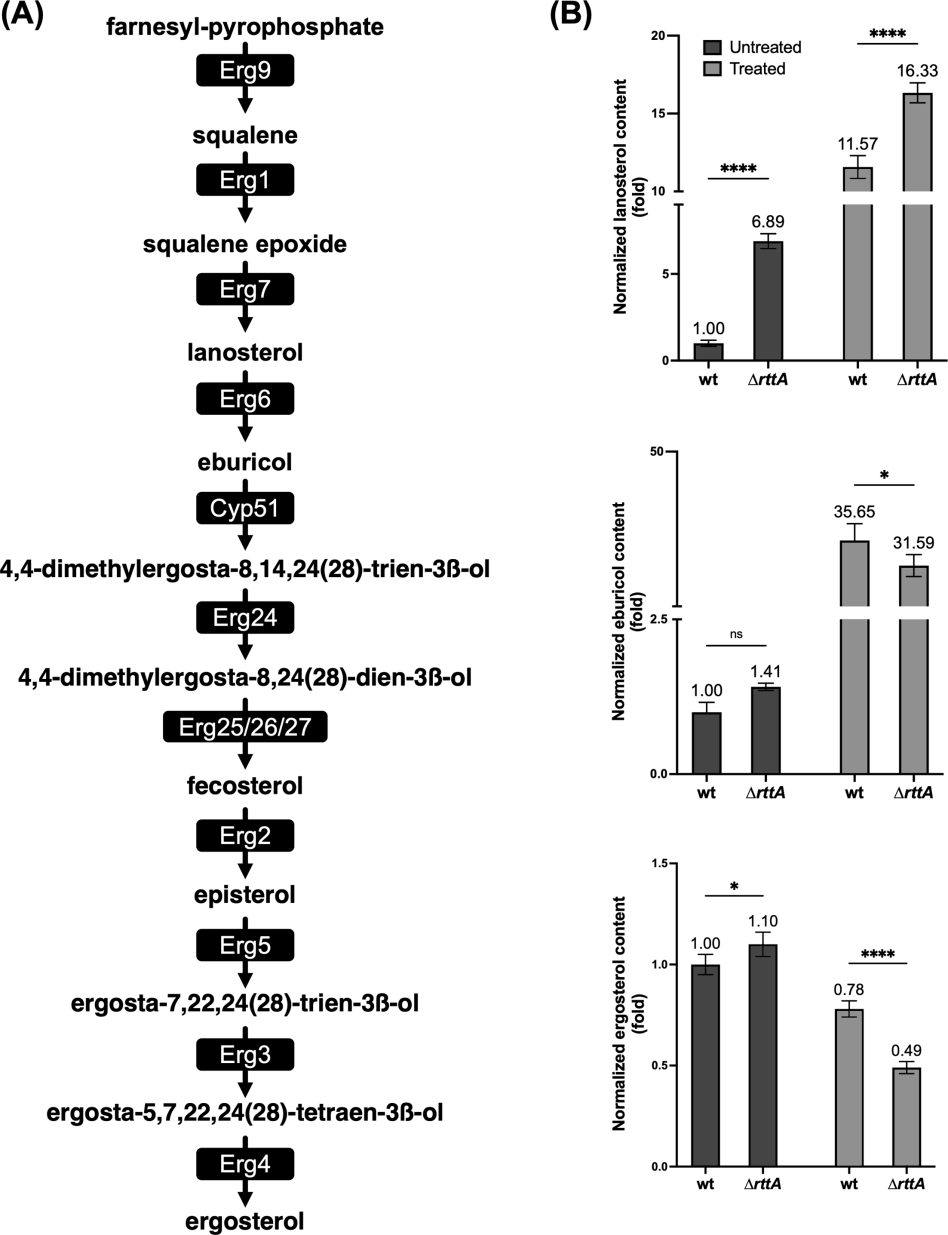
Lack of RttA leads to elevated lanosterol and decreased ergosterol levels upon azole exposure. (**A**) Schematic of the ergosterol biosynthesis pathway in *A. fumigatus*. (**B**) Relative levels of lanosterol, eburicol, and ergosterol (based on µg/mg biomass, Table S3) in ∆*rttA* compared to wild type (wt). For sterol analysis, strains were grown for 18 hours in AMM at 37°C in the presence (Treated) and absence (Untreated) of 0.06 µg/mL voriconazole. The respective amount of the solvent DMSO was used in the controls. Results represent the mean of biological triplicates, and error bars illustrate the standard deviation. *P*-values (pval) were calculated by two-way ordinary ANOVA. ****P < 0.0001, *P < 0.05, not significant (ns) *P* > 0.05.

As stated above, in *A. fumigatus,* lanosterol serves as a precursor for eburicol, the preferred substrate of sterol C14-demethylase Cyp51 in this species. The respective conversion is mediated by sterol C24-methyltransferase Erg6 (14, 18). Based on the idea that defective *erg6* regulation as a result of lack of *rttA* leads to changes in the sterol content, particularly lanosterol, the ergosterol biosynthesis intermediates were analyzed (Fig. 5 and Table S3). In addition to lanosterol, the content of the Cyp51 substrate eburicol and the final product ergosterol were determined in the presence and absence of voriconazole. In line with a specific reduction in *erg6* expression, the lanosterol content increased 6.9-fold in ∆*rttA* compared to wild type without azole treatment. Voriconazole exposure led to a significant increase (11.6-fold) of lanosterol in wild type, and the azole-induced accumulation of this intermediate was even more pronounced in ∆*rttA* (16.3-fold). Despite minor changes observed for the expression of *cyp51A* and *cyp51B* in ∆*rttA*, a slight increase in eburicol was observed in this mutant in the absence of voriconazole (1.4-fold). During voriconazole exposure, eburicol levels severely increased in wild type (35.7-fold) and were slightly higher than those observed for ∆*rttA* (31.6-fold). The content of the final product ergosterol, the by far most abundant sterol within the cell (see also absolute values in Table S3), was barely affected in ∆*rttA* without the addition of azoles. In the presence of voriconazole, however, ergosterol levels were substantially reduced.

### Upregulation of *erg6* partially recovers azole tolerance in ∆*rttA*

**Fig 6:**
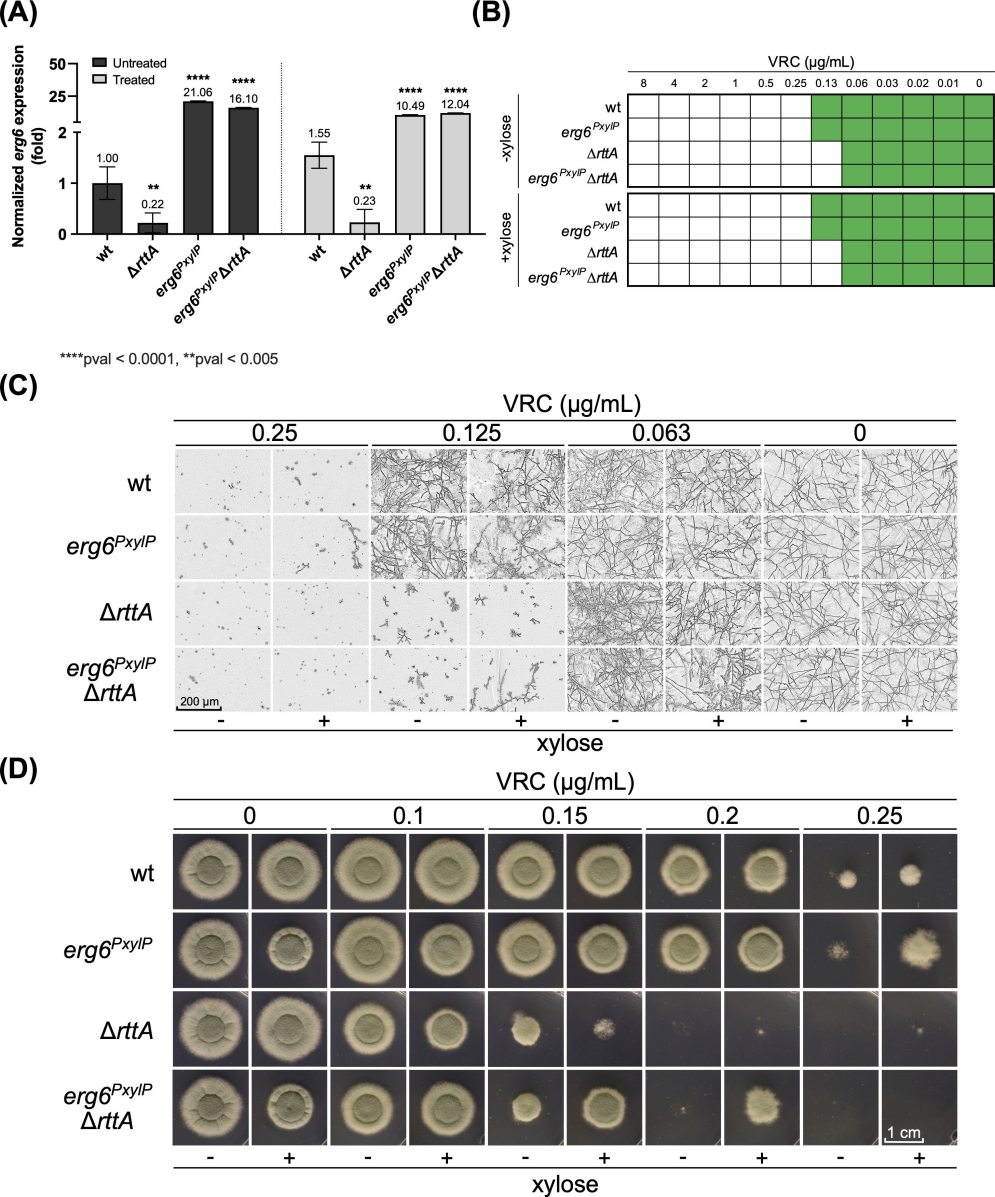
Induction of *erg6* partially restores azole tolerance. The effect of *erg6* upregulation on voriconazole (VRC) tolerance was tested in the background of wild type (strain *erg6^PxylP^*) and ∆*rttA* (strain *erg6^PxylP^*∆*rttA*). (**A**) RT-qPCR-based expression analysis of *erg6* in wild type (wt), ∆*rttA*, *erg6^PxylP^*, and *erg6^PxylP^*∆*rttA*. After pre-growth of strains in AMM for 17 hours at 37°C, 0.5 µg/mL voriconazole (Treated) was supplemented to the medium for a duration of 1 h. The respective amount of the solvent (DMSO, Untreated) was used in the controls. Results represent the mean of biological triplicates normalized to wild type (wt), and error bars illustrate the standard deviation. *P*-values (pval) were calculated by two-way ordinary ANOVA. Susceptibility testing was performed following the protocol according to EUCAST (57) in 96-well format (**B and C**) as well as on solid AMM (**D**) supplemented with different concentrations of voriconazole. Growth was monitored visually (**B and D**) as well as microscopically (**C**) after 48 hours. To induce *erg6* in *erg6^PxylP^* and *erg6^PxylP^*∆*rttA*, 1% xylose (+xylose) was added to the medium.

In previous works, downregulation of *erg6* could not be linked to increased azole susceptibility (14, 18). Nevertheless, to rule out whether *erg6* downregulation in ∆*rttA* could be connected to its altered susceptibility, we inserted a *PxylP*-inducible (55) *erg6* copy at the *fcyB* marker locus to conditionally upregulate the gene in the deletion mutant (strain *erg6^PxylP^*∆*rttA*) (56). As a further control, we also added the tunable *erg6* gene into the wild type (strain *erg6^PxylP^*). Expression of *erg6* during induction was validated by RT-qPCR analysis (Fig. 6A). In the 96-well-based broth microdilution assay, no difference in the MIC levels comparing background strains and the *erg6*-inducible mutants was detected (Fig. 6B). We observed enhanced growth during upregulation of *erg6* in ∆*rttA* in the presence of voriconazole, but only for individual hyphae (Fig. 6C; ∆*rttA* versus *erg6^PxylP^*∆*rttA*: 0.125 µg/mL voriconazole + 1% xylose). A similar effect was detected during induction of *erg6* in wild type (wild type versus *erg6^PxylP^*: 0.25 µg/mL voriconazole + 1% xylose). For multiple conidia or hyphae, no clear growth improvement was found.

In addition to the broth microdilution assay, we further performed susceptibility testing by monitoring growth on agar plates and observed a partial, but clear recovery of azole tolerance for *erg6^PxylP^*∆*rttA* in comparison to ∆*rttA* during *erg6* induction (Fig. 6D; ∆*rttA* versus *erg6^PxylP^*∆*rttA*: 0.1–0.25 µg/mL voriconazole + 1% xylose). Induction of *erg6* in the wild-type background also led to a slight growth improvement at the highest concentration of voriconazole tested (Fig. 6D, wild type versus *erg6^PxylP^*: 0.25 µg/mL voriconazole + 1% xylose).

## DISCUSSION

More than a decade ago, the *A. fumigatus* basic helix-loop-helix transcription factor SrbA was identified because its encoding gene was hypoxia-induced and the protein showed similarities to the previously described Sre1, the corresponding SREBP required for hypoxia adaptation in *S. pombe* (28, 60). The Zn_2_Cys_6_ binuclear zinc cluster transcription factor AtrR was originally discovered in a search for *A. oryzae* transcription factors with PDR1- and PDR3-related DNA binding domains (29). In the same study, the *A. fumigatus* AtrR homolog was elucidated and extensively investigated, revealing common gene targets with SrbA, but also unique targets including the azole efflux pump-encoding gene *cdr1B* (29, 31). PDR1 and its paralog PDR3 are transcription factors in *S. cerevisiae* that are involved in the regulation of pleiotropic drug response, including the expression of efflux pump-encoding genes known to promote azole resistance (61–64). Implying a potential regulatory nexus of RttA with both SrbA and AtrR, the latter two are crucial for adequate *rttA* expression. Notably, ChIP-seq analysis revealed *rttA* as a direct target of SrbA (35) but not AtrR (30), despite the presence of a putative AtrR consensus binding motif in the 5′ upstream region (CGGN_12_CCG, −228 to −245 relative to the translation start). The respective motif is located within the ChIP-seq spanning region (-92 to −356 relative to the translation start) detected for SrbA (35). As mentioned above, in *S. cerevisiae* and yeast pathogens *N. glabratus*, *C. albicans,* and *C. auris*, Upc2 homologs play crucial roles in the regulation of sterol biosynthesis genes, and Upc2 gain-of-function mutations were shown to drive azole resistance, at least in part due to increased expression of the azole drug target-encoding gene *ERG11* (39–47). SrbA and AtrR have overlapping regulatory functions with yeast Upc2, and to date, no Upc2 homolog has been described in *A. fumigatus* (65).

Here, we characterize the protein RttA as a further transcriptional regulator in *A. fumigatus* that participates in the regulation of ergosterol biosynthesis. *rttA*, among several other genes, has previously been linked to tolerance to the pesticide tebuconazole (36). In a further study (37), the gene was found to be upregulated along with several ergosterol biosynthesis and azole efflux pump encoding genes in azole persister cells (37). Considering the increase in resistance during *rttA* overexpression (Fig. 2, *rttA^PxylP^* + xylose), elevated *rttA* expression could have contributed to the observed persistence. Despite low similarity between the full-length version of RttA and yeast Upc2 homologs, our analyses suggested structural similarity of RttA and the respective yeast proteins at the C-terminus spanning the LBD (Fig. 1C). Even higher similarity was predicted with the putative LBD-covering region of *N. crassa* NcSR, which might indicate a closer relation of RttA to this protein. In *S. cerevisiae,* Upc2 and *N. glabratus* Upc2A, ergosterol was found to be the corresponding ligand that docks to the LBD that impedes its regulatory action by hindering its translocation to the nucleus (51, 52). Although our work might hint at Upc2 homologous functions of the RttA C-terminus in sterol binding, like for NcSR, further investigations and evidence will be required to uncover the specific ligand that dictates its activity.

A major difference in the enzymatic steps that lead to ergosterol biosynthesis in yeast illustrates Erg6-mediated conversion of lanosterol to eburicol before sterol C14-demethylation by Cyp51 in *A. fumigatus*. Yeast Erg6 acts in a later stage of the pathway, catalyzing the formation of fecosterol from zymosterol (12, 14, 18). Like in *A. fumigatus*, eburicol serves as a substrate of the sterol C14-demethylase Cyp51 homolog (Erg11) in *N. crassa* (38). The high structural similarity observed for RttA and NcSR, as well as analogous roles in *erg6* regulation insinuates an overlapping function of these proteins in sterol regulation. In contrast to *N. crassa erg11* being regulated by NcSR (38), however, the *A. fumigatus* orthologs *cyp51A* and *cyp51B* do not appear to be under the control of RttA. In *A. fumigatus,* adequate activation of *cyp51A* and *cyp51B* relies on functional SrbA and AtrR instead, and deletion mutants ∆*srbA* and ∆*atrR* display azole hypersusceptibility (26–30, 35). On the contrary, in *N. crassa* gene deletion mutants of the SREBP homolog SAH-2 and AtrR showed azole MIC levels like the respective wild type and *erg11* was not among their gene targets (38). Thus, NcSR seems to be the more critical regulator than those two proteins in *N. crassa* for azole adaptation, including *erg11* activation.

As stated above, in recent studies, downregulation of *erg6* could not be linked to increased azole susceptibility (14, 18); however, in ∆*rttA,* multiple genes are differentially expressed and, thus, the combined downregulation of several genes could account for its azole susceptibility. Notably, in one of the studies (18), a coinciding upregulation of azole resistance-associated efflux pumps was observed during *erg6* depletion, which might have counteracted azole toxicity (18). The absence of RttA leads to diminished *erg6* expression and consequently increased lanosterol levels and decreased ergosterol levels during azole treatment (Fig. 5B). This decrease in ergosterol is most likely one contributing factor to the azole susceptibility observed for ∆*rttA*. Overexpression of *erg6* in ∆*rttA* led to a partial but clear increase in azole tolerance (Fig. 6D). In addition to *erg6*, expression analysis (Fig. 4) revealed a positive regulatory role of RttA on fungal efflux. *abcA* (59), its neighbor gene *AFUB_030800* encoding a putative MFS drug transporter and *cdr1B* (31) were downregulated in *∆rttA* and upregulated during *rttA* induction upon azole exposure. These outcomes suggest that altered resistance in the *rttA* mutant strains involves multiple factors, most likely differential expression of *erg6* and genes coding for efflux pumps.

Our study collectively identifies RttA as a Zn_2_Cys_6_ binuclear zinc cluster transcription factor that participates in *A. fumigatus* sterol homeostasis by regulating *erg6*. In addition to *erg6*, RttA plays a crucial role in the expression of azole resistance-associated efflux pump-encoding genes. Its structural similarities to yeast Upc2 and *N. crassa* NcSR indicate a similar mechanism controlling their regulatory activity and remain an important avenue for future research.

## MATERIALS AND METHODS

### Minimum inhibitory concentration testing

Minimum inhibitory concentrations (MICs) were determined following the procedure described by EUCAST (57) using RPMI-1640 medium (Sigma-Aldrich, St. Louis, MO, USA). To induce genes under the control of *PxylP*, 1% (wt/vol) xylose was added to the medium. MICs were visually assessed after 48 hours of incubation at 37°C.

### Radial growth assay

Radial growth of strains was monitored using solid AMM (66) containing 20 mM ammonium tartrate as the nitrogen source, 1% (wt/vol) glucose as the carbon source, and 1.5% (wt/vol) agar. 10^4^ spores in a total volume of 5 µL of spore buffer (0.1% (vol/vol) Tween 20 and 0.9% (wt/vol) NaCl in H_2_O) were point inoculated onto solid AMM. Plates were incubated for 48 hours at 37°C before image acquisition. Growth phenotypes of strains were assessed in the presence of voriconazole or during hypoxic conditions that were adjusted to 1% O_2_, 5% CO_2_, 94% N_2_ (C-Chamber and Pro-Ox, Pro-CO_2_ controller; Biospherics) (67).

### Generation of fungal mutants

The strains used in this study are listed in Table S4, and the primers used are listed in Table S5. To generate the deletion mutant Δ*rttA*, the 5′ and 3′ flanking regions (approximately 1 kb) of the *rttA* gene were PCR amplified from genomic DNA of *A. fumigatus* using the primer pairs rttA-1/2 and rttA-3/4. The hygromycin B resistance cassette was amplified from the pAN7-1 plasmid (68) using the hph-FW/RV primer pair. 5′ and 3′ flanking regions were subsequently connected *via* fusion PCR as described in previous work (31) employing nested primers rttA-N1/N2. The yielding deletion cassette (Fig. S2) was then used to transform *A. fumigatus* A1160P+ (31), which was used as a background strain in this work.

To generate overexpression mutants of *rttA*, *atrR*, *srbA,* and *erg6*, a linear backbone containing *PxylP* was amplified from pΔfcyB_cyp51A^PxylP^ using the pX-FW.2/RV.2 primer pair as previously described (15). The *rttA*, *atrR*, *srbA,* and *erg6* coding sequences were amplified from genomic DNA using rttA-FW/RV, atrR-FW/RV, srbA-FW/RV, and erg6-FW/RV primer pairs, respectively. The plasmid backbone was then individually assembled with the coding sequences carrying overlaps to the backbone using the NEBuilder HiFi DNA Assembly Master Mix (New England Biolabs Inc., Ipswich, MA, USA). The yielding plasmids p∆fcyB-rttA^PxylP^, p∆fcyB-atrR^PxylP^, p∆fcyB-srbA^PxylP^, and p∆fcyB-erg6^PxylP^ (Fig. S2) were linearized with *Not*I before being used for transformation of *A. fumigatus* A1160P+. *Not*I-linearized pΔfcyB-erg6^PxylP^ plasmid was further used to transform Δ*rttA*.

To reconstitute the *rttA* deletion mutant, plasmid pSK275-rttA^REC^ was generated. For this purpose, pSK275 was amplified using the BBpSK275-FW/RV primer pair, yielding a linear backbone that contained the pyrithiamine resistance gene. The *rttA* gene sequence was amplified from the genomic DNA of *A. fumigatus* using the rttArecon-FW/RV primer pair. The two DNA fragments were then assembled as described above. The resulting plasmid was *Bgl*II linearized and used to transform the Δ*rttA A. fumigatus* background (Fig. S2). Fungal transformations using selection agents 5-fluorocytosine, hygromycin B, and pyrithiamine were carried out as previously described (26, 56). All strains generated in this work were validated by PCR (Fig. S3).

### Expression analyses

For expression analysis, conical 500 mL flasks containing 100 mL of AMM inoculated with 10^8^ spores were incubated at 37°C in an orbital shaker at 200 rpm. For RNA-seq analysis, strains were grown for 18 hours. For RT-qPCR analysis (Fig. 4 and 6), strains were first incubated for 17 hours and, subsequently, short-term azole-stress was induced by adding 0.5 µg/mL voriconazole to the medium for 1 hour. As a no-drug control, the same amount of the respective solvent (DMSO) was supplemented to the medium. To induce *rttA* in strain *rttA^PxylP^* and *erg6* in *erg6^PxylP^*, as well as *erg6^PxylP^*∆*rttA*, 1% (wt/vol) xylose was directly added to the cultures. After incubation, biomass was collected by filtration, shock-frozen, and freeze-dried. 10 mg freeze-dried and pulverized mycelium was used for RNA extraction. Total RNA was extracted using 1 mL TRI Reagent (Sigma-Aldrich, St. Louis, MO, USA) according to the manufacturer’s recommendations. For RNA-seq and RT-qPCR analysis, 10 µg of total RNA was digested using RQ1 RNAse-Free DNase (Promega Corp., Madison, WI, USA) and further purified using the Monarch RNA Cleanup Kit (New England Biolabs Inc., Ipswich, MA, USA). cDNA for reverse RT-qPCR analysis was subsequently synthesized from 500 ng of total purified RNA using the LunaScript RT Supermix (New England Biolabs Inc., Ipswich, MA, USA). RT-qPCR was performed using the SYBR Green-based Luna Universal qPCR chemistry (New England Biolabs Inc., Ipswich, MA, USA) on a QuantStudio 3 Light Cycler (Applied Biosystems Inc., Carlsbad, CA, USA). The primers used for RT-qPCR are listed in Table S5. Amplification reactions were carried out in a final volume of 10 µL using 1 ng of total RNA and 0.25 µM of each forward and reverse primer. For RNA-seq analysis, Poly-(A)-tailed mRNA was enriched, and directional sequencing of mRNA was performed on a NovaSeq X Plus platform (Novogene GmbH, Planegg, Germany) using a paired-end 150 bp strategy. Raw sequencing reads were processed using nf-core/rnaseq (v3.17.0; https://doi.org/10.5281/zenodo.1400710) of the nf-core collection of workflows (69), utilizing reproducible software environments provided by Singularity (70). Briefly, reads underwent quality control and adapter trimming prior to alignment to the *Aspergillus fumigatus* A1163 genome (FungiDB, release 68) (71) using STAR (v2.7.11b), followed by quantification with Salmon (v1.10.3). Alignments were sorted and indexed with Samtools (v1.21) and visualized in IGV (v2.16.0; (72)). Differential expression analysis was carried out using the Bioconductor package DESeq2 (v1.49.0) in R (v4.5.0). Filtering and plotting were performed using the R packages dplyr (v1.1.4), ggplot2 (v3.5.2), and ComplexHeatmap (v2.25.0). The integrative genomics viewer (IGV) (72) was used to visualize mapped RNA-seq reads to the annotated *rttA* gene and correct its coding sequence.

All experiments were performed in triplicate.

### Quantification of sterols and voriconazole

For sterol analysis, conical 500 mL flasks containing 100 mL of AMM, with and without 0.06 µg/mL voriconazole, were inoculated with 10^8^ spores and incubated at 37°C in an orbital shaker at 200 rpm. Sterol analysis was performed as previously described using 6 mg of pulverized mycelium (73).

## Data Availability

All next-generation sequencing data are available through the BioProject PRJNA1274688.

## SUPPLEMENTAL MATERIAL

Supplemental Figures and Tables mbio.01854-25-s0001.pdf Fig. S1 to S3; Tables S1, S4, and S5.

Table S2 mbio.01854-25-s0002.xlsx Differentially expressed genes in ∆*rttA*, ∆*srbA*, and ∆*atrR* versus wild type.

Table S3 mbio.01854-25-s0003.xlsx Sterol levels of wt and ∆*rttA*.

ASM does not own the copyrights to Supplemental Material that may be linked to, or accessed through, an article. The authors have granted ASM a non-exclusive, world-wide license to publish the Supplemental Material files. Please contact the corresponding author directly for reuse.
