# Supplementary material for: The transcription factor RttA contributes to sterol regulation and azole resistance in Aspergillus fumigatus
Source: mBio. 2025 Sep 12;16(10):e01854-25. doi: 10.1128/mbio.01854-25 (PMC12505884; doi:10.1128/mbio.01854-25)
Supplement: Supplemental Figures and Tables — Fig. S1 to S3; Tables S1, S4, and S5. [file mbio.01854-25-s0001.pdf]

Fig S1 **MUSCLE-based multiple sequence alignment of RttA, NcSR and yeast Upc2 homologs.** The alignment was used to predict the peptide regions of each protein that overlaps the Zn_2Cys_6 binuclear cluster and the C-terminus containing the potential ligand binding domain. The sequence conservation is shown as grey bars. The consensus motif threshold was put to 95%. Amino acids are colored based on their properties and conservation (ClustalX).

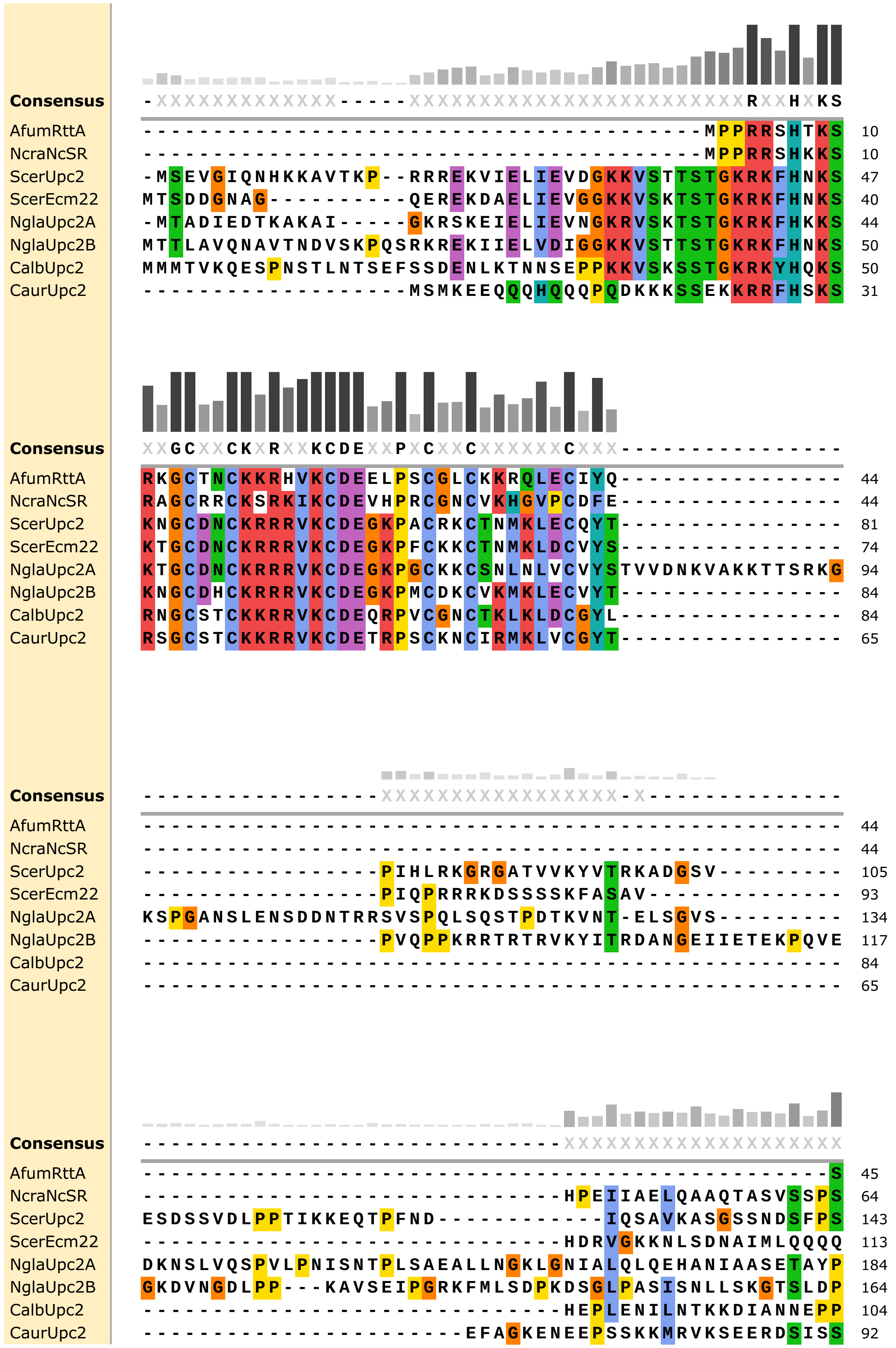

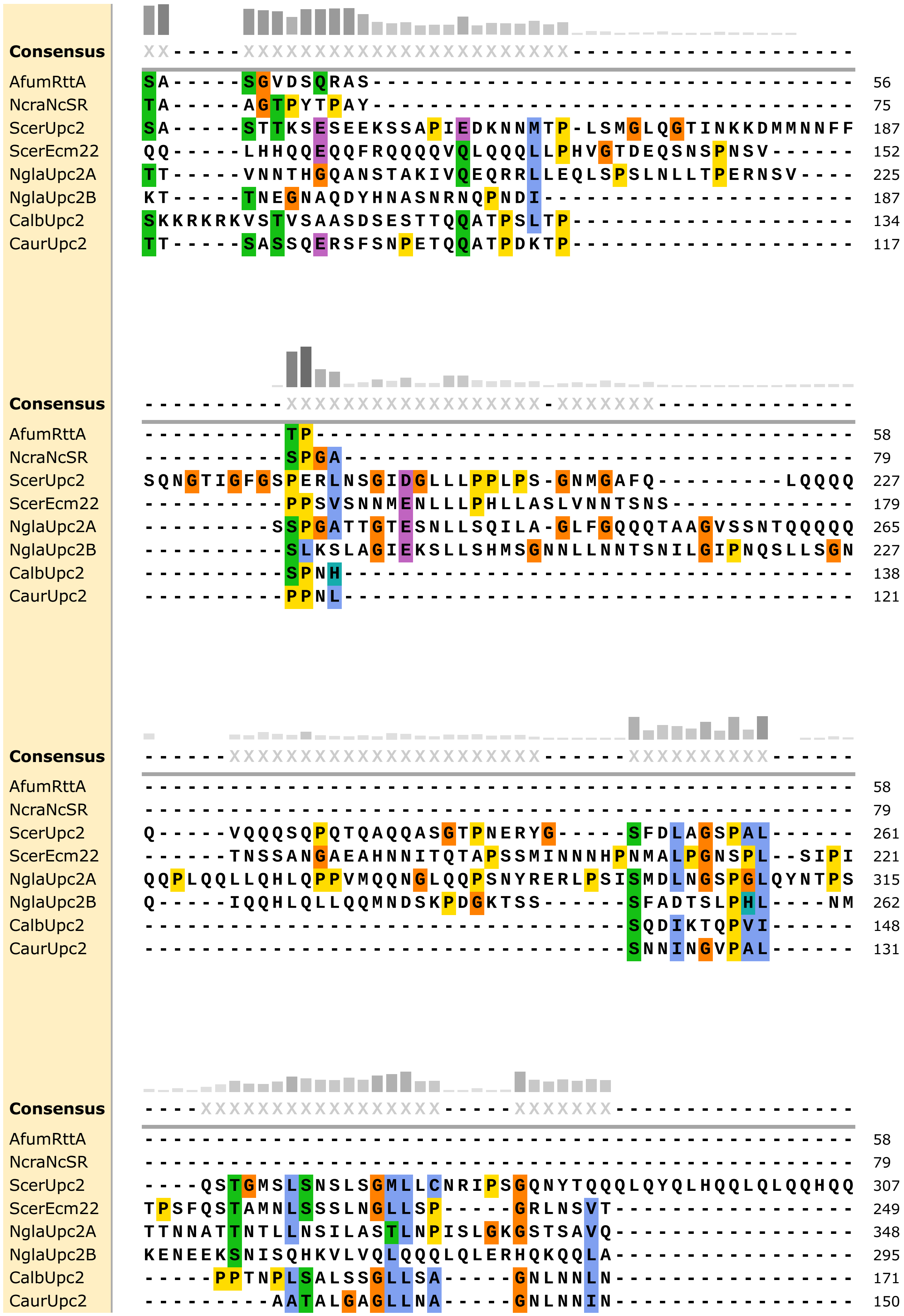
Consensus XX-----XXXXXXXXXXXXXXXXXXXXXXX--------------------
AfumRttA SA-----SGVDSQRAS---------------------------------- 56
NcraNcSR TA-----AGTPYTPAY---------------------------------- 75
ScerUpc2 SA-----STTKSESEEKSSAPIEDKNNMTP-LSMGLQGTINKKDMMNNFF 187
ScerEcm22 QQ-----LHHQQEQQFRQQQQVQLQQQLLPHVGTDEQSNSPNSV------ 152
NglaUpc2A TT-----VNNTHGQANSTAKIVQEQRRLLEQLSPSLNLLTPERNSV---- 225
NglaUpc2B KT-----TNEGNAQDYHNASNRNQPNDI---------------------- 187
CalbUpc2 SKKRKRKVSTVSAASDSESTTQQATPSLTP-------------------- 134
CaurUpc2 TT-----SASSQERSFSNPETQQATPDKTP-------------------- 117
Consensus ----------XXXXXXXXXXXXXXXXXX-XXXXXXX--------------
AfumRttA ----------TP-------------------------------------- 58
NcraNcSR ----------SPGA------------------------------------ 79
ScerUpc2 SQNGTIGFGSPERLNSGIDGLLLPPLPS-GNMGAFQ---------LQQQQ 227
ScerEcm22 ----------PPSVSNNMENLLLPHLLASLVNNTSNS------------- 179
NglaUpc2A ---------SSPGATTGTESNLLSQILA-GLFGQQQTAAGVSSNTQQQQQ 265
NglaUpc2B ----------SLKSLAGIEKSLLSHMSGNNLLNNTSNILGIPNQSLLSGN 227
CalbUpc2 ----------SPNH------------------------------------ 138
CaurUpc2 ----------PPNL------------------------------------ 121
Consensus ------XXXXXXXXXXXXXXXXXXXXXX------XXXXXXXXXX------
AfumRttA -- 58
NcraNcSR -- 79
ScerUpc2 Q-----VQQQSQPQTQAQQASGTPNERYG-----SFDLAGSPAL------ 261
ScerEcm22 ------TNSSANGAEAHNNITQTAPSSMINNNHPNMALPGNSPL--SIPI 221
NglaUpc2A QQPLQQLLQHLQPPVMQQNGLQQPSNYRERLPSISMDLNGSPGLQYNTPS 315
NglaUpc2B Q-----IQQHLQLLQQMNDSKPDGKTSS------SFADTSLPHL----NM 262
CalbUpc2 ----------------------------------SQDIKTQPVI------ 148
CaurUpc2 ----------------------------------SNNINGVPAL------ 131
Consensus ----XXXXXXXXXXXXXXXXX-----XXXXXXX-----------------
AfumRttA -- 58
NcraNcSR -- 79
ScerUpc2 ----QSTGMSLSNSLSGMLLCNRIPSGQNYTQQQLQYQLHQQLQLQQHQQ 307
ScerEcm22 TPSFQSTAMNLSSSLNGLLSP-----GRLNSVT----------------- 249
NglaUpc2A TTNNATTNTLLNSILASTLNPISLGKGSTSAVQ----------------- 348
NglaUpc2B KENEEKSNISQHKVLVQLQQQLQLERHQKQQLA----------------- 295
CalbUpc2 -----PPTNPLSALSSGLLSA-----GNLNNLN----------------- 171
CaurUpc2 ---------AATALGAGLLNA-----GNLNNIN----------------- 150

```
Consensus   --------------------------------XXXXXXXXXXXXXXXXXX
AfumRttA    --------------------------------------------------  58
NcraNcSR    --------------------------------------------------  79
ScerUpc2    VQLQQYQQLRQEQHQQVQQQQQEQLQQYQQHFLQQQQQVLLQQEQQPNDE  357
ScerEcm22   ---------------------------------NGLQQPQLQQQNQQIPQ  266
NglaUpc2A   ---------------------------------ANSSDGLMGATSTRNED  365
NglaUpc2B   ----------QYQKLQMEQQQEFIKRSQEHENSSSSKSSLAAESVSSYDE  335
CalbUpc2    --------------------------------VAHLVNNLSGLGDLSNLA  189
CaurUpc2    --------------------------------LSHLVNNLSNLGDLSSLG  168
```

```
Consensus   XXXXX----------------------XXXXXXXXXXXXXXXXXXX--XX
AfumRttA    --------------------------------------------------  58
NcraNcSR    --------------------------------------------------  79
ScerUpc2    EGGVQEENSKKVKEGPLQSQTSETTLNSDAATLQADALSQLSKMGL--SL  405
ScerEcm22   QQGTQ----------------------SPFSNIPFDQLAQLNKMGLNFNM  294
NglaUpc2A   SIGNA----------------------LPFST---DAISQLTKLNL--NS  388
NglaUpc2B   QKGKIDNNN------------------NSATAVQADTLAQLSKLGI--SM  365
CalbUpc2    SLGNL---------------------------ASLSNLASLAQLPI--DL  210
CaurUpc2    NVANL------------------------------SSLAVLAQLPI----  184
```

```
Consensus   XXXXX-XXXXXXXXXXXXXXXXXXXXXX--XXXXXXXXXXXXXXXXXXXX
AfumRttA    --------------------------------------------REMIDT  64
NcraNcSR    ------------------------------ESVSNASTSRFQRAPPLCRS  99
ScerUpc2    KSLST-FPTAGIGGVSYDFQELLGI-KFPINNGNSRATKASNAEEALANM  453
ScerEcm22   KSFNTLFPYGAANGMASEFQELFGLGKF--ATSNNRAIKVSTAEEALANM  342
NglaUpc2A   ------FPTAGIGGISYDFHELFGI-KY--NHTNNRAIKVSSAEEALANM  429
NglaUpc2B   KGLSS-LLTAGFGGVQYDFQALLGLKNL----GQSKQDKANYAHDALTSM  410
CalbUpc2    SNLGSLLDSPAASNIAASFLGSAAATTV--PPTTNSEFKESNQRKSQTQM  258
CaurUpc2    ----------DLSGLSNQFAGGFDGSMGNANGNLQGNARINQSAPMPQQA  224
```

```
Consensus   XXXXXX-------------XXXXXXXXXXXXXXXXXXXXXXXXXXXXXXX
AfumRttA    KPQEW---------------------------------------------  69
NcraNcSR    PPSQAL--------------------------------------------  105
ScerUpc2    QEHHER-------------AAASVKENDGQLSDTKSPAPSNNAQGGSASI  490
ScerEcm22   QQEQEDKNKQFTKNPLDNTKTDAVNSGNNPLNGNENKVTASDILSHNKNL  392
NglaUpc2A   QEHREREQASKISEKQKQAAEQETRNSENGVINKQNSIGINASSENSVSA  479
NglaUpc2B   QEDHEY------------ARDEKIKMETTANNDINRMLGSSN--------  440
CalbUpc2    PPQPTV-------------PITSMGAATTTSSHQQANMPSRSKPQPETLQ  295
CaurUpc2    IPSQEK--------------------------------------------  230
```

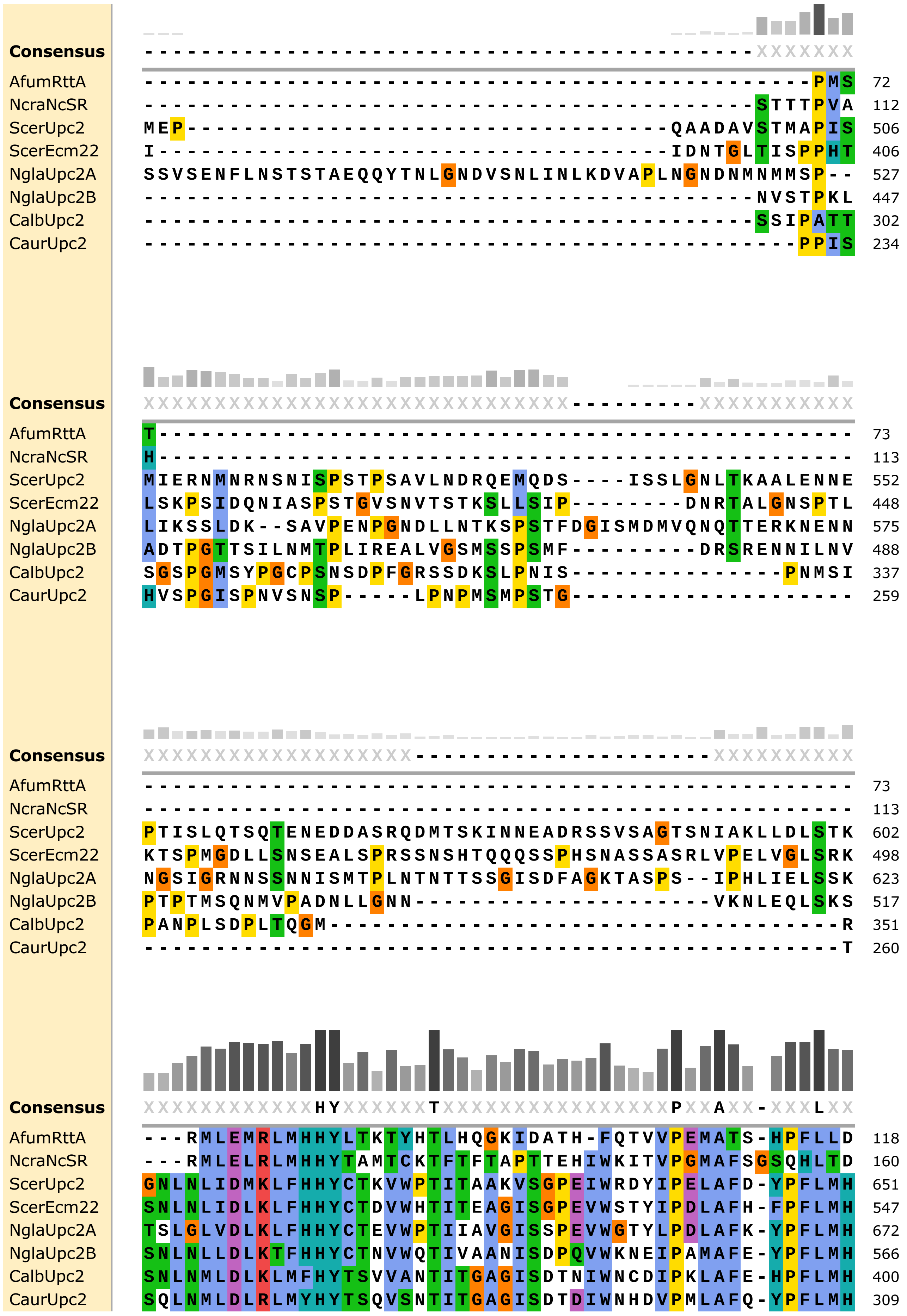
Consensus ---XXXXXXX
AfumRttA ---PMS 72
NcraNcSR ---STTTPVA 112
ScerUpc2 MEP----------------------------------QAADAVSTMAPIS 506
ScerEcm22 I------------------------------------IDNTGLTISPPHT 406
NglaUpc2A SSVSENFLNSTSTAEQQYTNLGNDVSNLINLKDVAPLNGNDNMNMMSP-- 527
NglaUpc2B ---NVSTPKL 447
CalbUpc2 ---SSIPATT 302
CaurUpc2 --PPIS 234
Consensus XXXXXXXXXXXXXXXXXXXXXXXXXXXXXX---------XXXXXXXXXXX
AfumRttA T--- 73
NcraNcSR H--- 113
ScerUpc2 MIERNMNRNSNISPSTPSAVLNDRQEMQDS----ISSLGNLTKAALENNE 552
ScerEcm22 LSKPSIDQNIASPSTGVSNVTSTKSLLSIP--------DNRTALGNSPTL 448
NglaUpc2A LIKSSLDK--SAVPENPGNDLLNTKSPSTFDGISMDMVQNQTTERKNENN 575
NglaUpc2B ADTPGTTSILNMTPLIREALVGSMSSPSMF---------DRSRENNILNV 488
CalbUpc2 SGSPGMSYPGCPSNSDPFGRSSDKSLPNIS---------------PNMSI 337
CaurUpc2 HVSPGISPNVSNSP-----LPNPMSMPSTG-------------------- 259
Consensus XXXXXXXXXXXXXXXXXXX---------------------XXXXXXXXXX
AfumRttA -- 73
NcraNcSR -- 113
ScerUpc2 PTISLQTSQTENEDDASRQDMTSKINNEADRSSVSAGTSNIAKLLDLSTK 602
ScerEcm22 KTSPMGDLLSNSEALSPRSSNSHTQQQSSPHSNASSASRLVPELVGLSRK 498
NglaUpc2A NGSIGRNNSSNNISMTPLNTNTTSSGISDFAGKTASPS--IPHLIELSSK 623
NglaUpc2B PTPTMSQNMVPADNLLGNN---------------------VKNLEQLSKS 517
CalbUpc2 PANPLSDPLTQGM------------------------------------R 351
CaurUpc2 ---T 260
Consensus XXXXXXXXXXXXHYXXXXXXTXXXXXXXXXXXXXXXXPXXAXX-XXXLXX
AfumRttA ---RMLEMRLMHHYLTKTYHTLHQGKIDATH-FQTVVPEMATS-HPFLLD 118
NcraNcSR ---RMLELRLMHHYTAMTCKTFTFTAPTTEHIWKITVPGMAFSGSQHLTD 160
ScerUpc2 GNLNLIDMKLFHHYCTKVWPTITAAKVSGPEIWRDYIPELAFD-YPFLMH 651
ScerEcm22 SNLNLIDLKLFHHYCTDVWHTITEAGISGPEVWSTYIPDLAFH-FPFLMH 547
NglaUpc2A TSLGLVDLKLFHHYCTEVWPTIIAVGISSPEVWGTYLPDLAFK-YPFLMH 672
NglaUpc2B SNLNLLDLKTFHHYCTNVWQTIVAANISDPQVWKNEIPAMAFE-YPFLMH 566
CalbUpc2 SNLNMLDLKLMFHYTSVVANTITGAGISDTNIWNCDIPKLAFE-HPFLMH 400
CaurUpc2 SQLNMLDLRLMYHYTSQVSNTITGAGISDTDIWNHDVPMLAFQ-YPFLMH 309

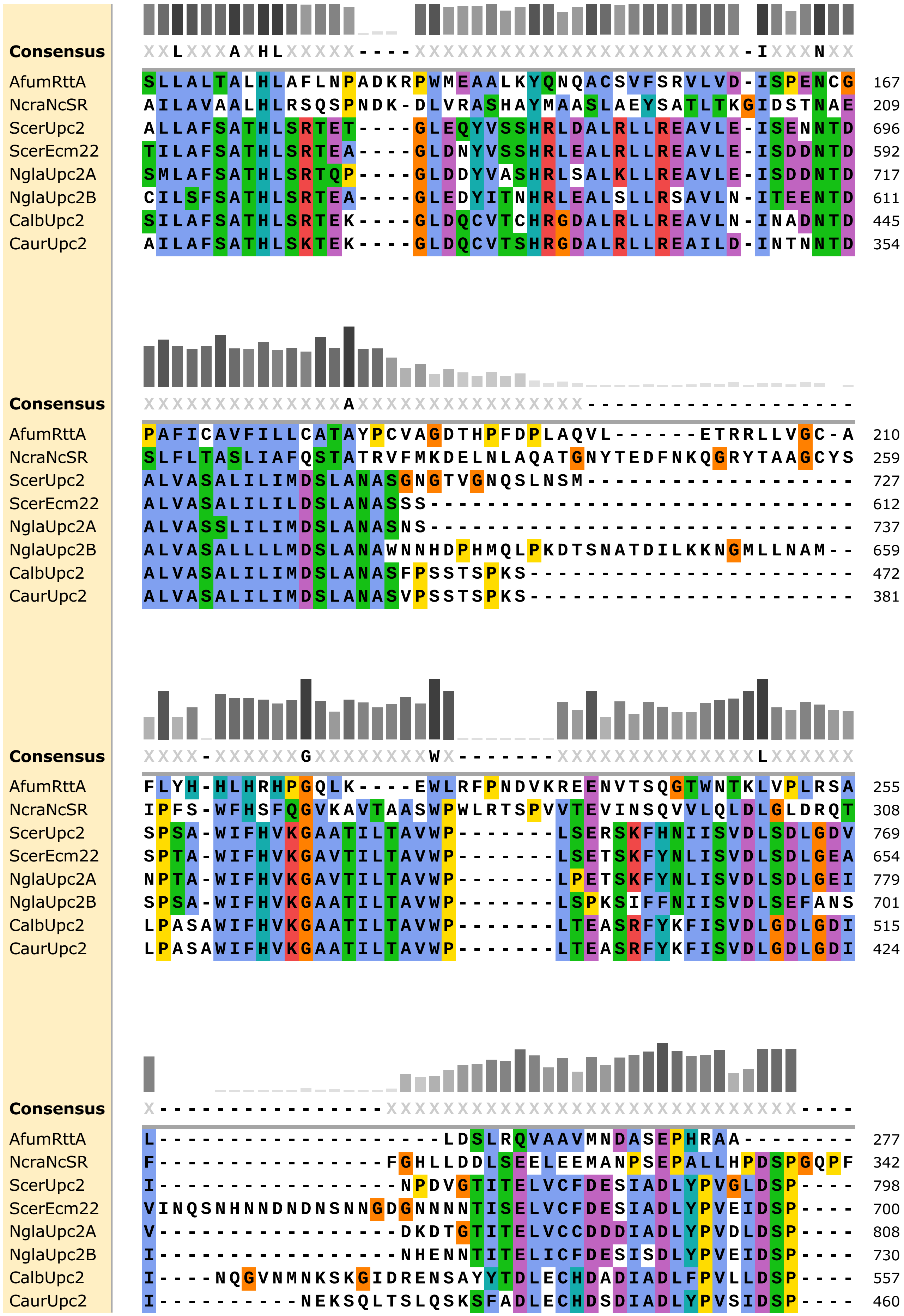
Consensus XXLXXXAXHLXXXXX----XXXXXXXXXXXXXXXXXXXXXXX-IXXXNXX
AfumRttA SLLALTALHLAFLNPADKRPWMEAALKYQNQACSVFSRVLVD-ISPENCG 167
NcraNcSR AILAVAALHLRSQSPNDK-DLVRASHAYMAASLAEYSATLTKGIDSTNAE 209
ScerUpc2 ALLAFSATHLSRTET----GLEQYVSSHRLDALRLLREAVLE-ISENNTD 696
ScerEcm22 TILAFSATHLSRTEA----GLDNYVSSHRLEALRLLREAVLE-ISDDNTD 592
NglaUpc2A SMLAFSATHLSRTQP----GLDDYVASHRLSALKLLREAVLE-ISDDNTD 717
NglaUpc2B CILSFSATHLSRTEA----GLEDYITNHRLEALSLLRSAVLN-ITEENTD 611
CalbUpc2 SILAFSATHLSRTEK----GLDQCVTCHRGDALRLLREAVLN-INADNTD 445
CaurUpc2 AILAFSATHLSKTEK----GLDQCVTSHRGDALRLLREAILD-INTNNTD 354
Consensus XXXXXXXXXXXXXXAXXXXXXXXXXXXXXXX-------------------
AfumRttA PAFICAVFILLCATAYPCVAGDTHPFDPLAQVL------ETRRLLVGC-A 210
NcraNcSR SLFLTASLIAFQSTATRVFMKDELNLAQATGNYTEDFNKQGRYTAAGCYS 259
ScerUpc2 ALVASALILIMDSLANASGNGTVGNQSLNSM------------------- 727
ScerEcm22 ALVASALILILDSLANASSS------------------------------ 612
NglaUpc2A ALVASSLILIMDSLANASNS------------------------------ 737
NglaUpc2B ALVASALLLLMDSLANAWNNHDPHMQLPKDTSNATDILKKNGMLLNAM-- 659
CalbUpc2 ALVASALILIMDSLANASFPSSTSPKS----------------------- 472
CaurUpc2 ALVASALILIMDSLANASVPSSTSPKS----------------------- 381
Consensus XXXX-XXXXXXGXXXXXXXXWX-------XXXXXXXXXXXXXXLXXXXXX
AfumRttA FLYH-HLHRHPGQLK----EWLRFPNDVKREENVTSQGTWNTKLVPLRSA 255
NcraNcSR IPFS-WFHSFQGVKAVTAASWPWLRTSPVVTEVINSQVVLQLDLGLDRQT 308
ScerUpc2 SPSA-WIFHVKGAATILTAVWP-------LSERSKFHNIISVDLSDLGDV 769
ScerEcm22 SPTA-WIFHVKGAVTILTAVWP-------LSETSKFYNLISVDLSDLGEA 654
NglaUpc2A NPTA-WIFHVKGAVTILTAVWP-------LPETSKFYNLISVDLSDLGEI 779
NglaUpc2B SPSA-WIFHVKGAATILTAVWP-------LSPKSIFFNIISVDLSEFANS 701
CalbUpc2 LPASAWIFHVKGAATILTAVWP-------LTEASRFYKFISVDLGDLGDI 515
CaurUpc2 LPASAWIFHVKGAATILTAVWP-------LTEASRFYKFISVDLGDLGDI 424
Consensus X----------------XXXXXXXXXXXXXXXXXXXXXXXXXXXXX----
AfumRttA L--------------------LDSLRQVAAVMNDASEPHRAA-------- 277
NcraNcSR F----------------FGHLLDDLSEELEEMANPSEPALLHPDSPGQPF 342
ScerUpc2 I-----------------NPDVGTITELVCFDESIADLYPVGLDSP---- 798
ScerEcm22 VINQSNHNNDNDNSNNGDGNNNNTISELVCFDESIADLYPVEIDSP---- 700
NglaUpc2A V-----------------DKDTGTITELVCCDDDIADLYPVDLDSP---- 808
NglaUpc2B I-----------------NHENNTITELICFDESISDLYPVEIDSP---- 730
CalbUpc2 I----NQGVNMNKSKGIDRENSAYYTDLECHDADIADLFPVLLDSP---- 557
CaurUpc2 I----------NEKSQLTSLQSKSFADLECHDSDIADLYPVSIDSP---- 460

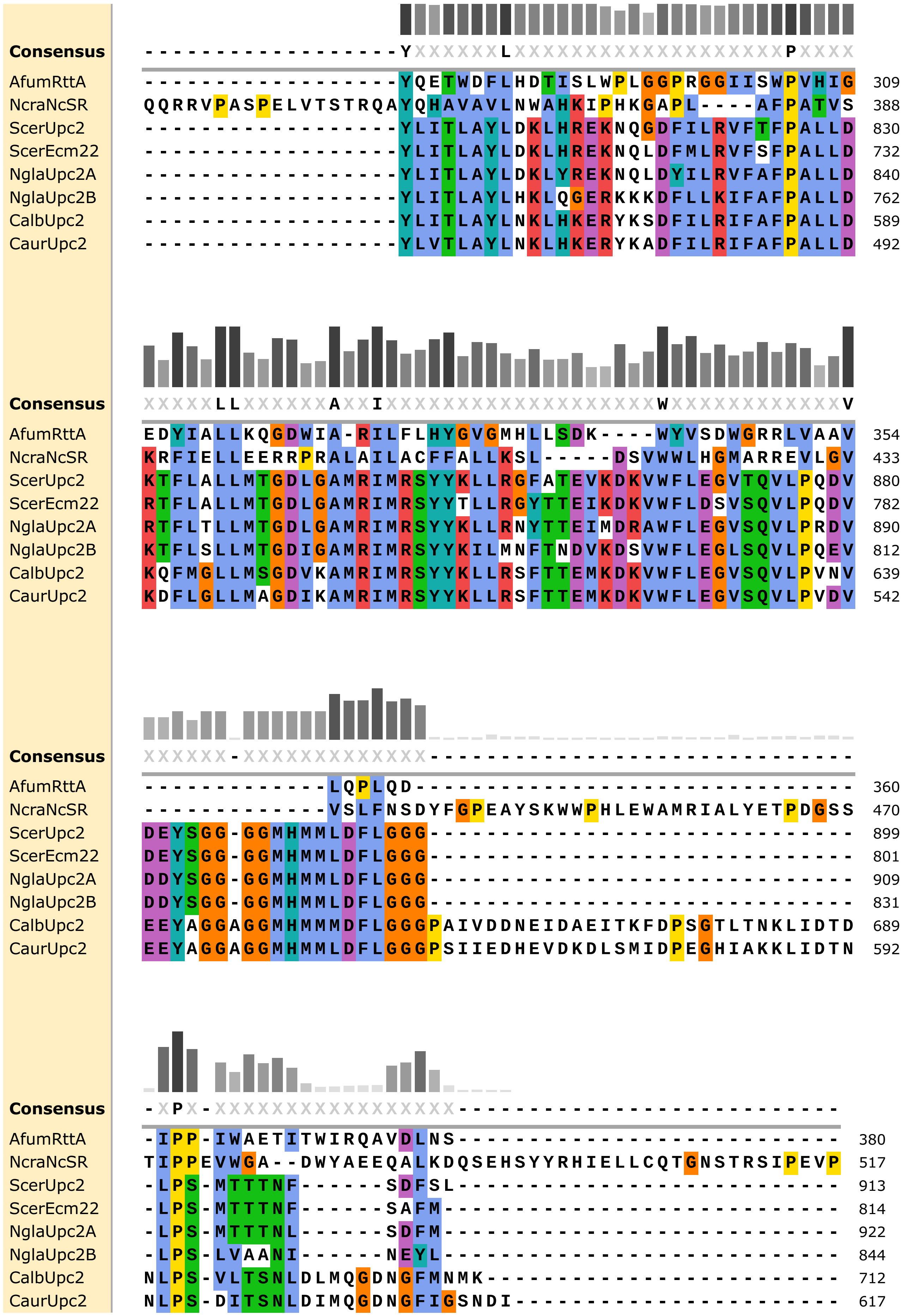
Consensus ------------------YXXXXXXLXXXXXXXXXXXXXXXXXXXPXXXX
AfumRttA ------------------YQETWDFLHDTISLWPLGGPRGGIISWPVHIG 309
NcraNcSR QQRRVPASPELVTSTRQAYQHAVAVLNWAHKIPHKGAPL----AFPATVS 388
ScerUpc2 ------------------YLITLAYLDKLHREKNQGDFILRVFTFPALLD 830
ScerEcm22 ------------------YLITLAYLDKLHREKNQLDFMLRVFSFPALLD 732
NglaUpc2A ------------------YLITLAYLDKLYREKNQLDYILRVFAFPALLD 840
NglaUpc2B ------------------YLITLAYLHKLQGERKKKDFLLKIFAFPALLD 762
CalbUpc2 ------------------YLITLAYLNKLHKERYKSDFILRIFAFPALLD 589
CaurUpc2 ------------------YLVTLAYLNKLHKERYKADFILRIFAFPALLD 492
Consensus XXXXXLLXXXXXXAXXIXXXXXXXXXXXXXXXXXXXWXXXXXXXXXXXXV
AfumRttA EDYIALLKQGDWIA-RILFLHYGVGMHLLSDK----WYVSDWGRRLVAAV 354
NcraNcSR KRFIELLEERRPRALAILACFFALLKSL-----DSVWWLHGMARREVLGV 433
ScerUpc2 KTFLALLMTGDLGAMRIMRSYYKLLRGFATEVKDKVWFLEGVTQVLPQDV 880
ScerEcm22 RTFLALLMTGDLGAMRIMRSYYTLLRGYTTEIKDKVWFLDSVSQVLPQDV 782
NglaUpc2A RTFLTLLMTGDLGAMRIMRSYYKLLRNYTTEIMDRAWFLEGVSQVLPRDV 890
NglaUpc2B KTFLSLLMTGDIGAMRIMRSYYKILMNFTNDVKDSVWFLEGLSQVLPQEV 812
CalbUpc2 KQFMGLLMSGDVKAMRIMRSYYKLLRSFTTEMKDKVWFLEGVSQVLPVNV 639
CaurUpc2 KDFLGLLMAGDIKAMRIMRSYYKLLRSFTTEMKDKVWFLEGVSQVLPVDV 542
Consensus XXXXXX-XXXXXXXXXXXXX------------------------------
AfumRttA -------------LQPLQD------------------------------- 360
NcraNcSR -------------VSLFNSDYFGPEAYSKWWPHLEWAMRIALYETPDGSS 470
ScerUpc2 DEYSGG-GGMHMMLDFLGGG------------------------------ 899
ScerEcm22 DEYSGG-GGMHMMLDFLGGG------------------------------ 801
NglaUpc2A DDYSGG-GGMHMMLDFLGGG------------------------------ 909
NglaUpc2B DDYSGG-GGMHMMLDFLGGG------------------------------ 831
CalbUpc2 EEYAGGAGGMHMMMDFLGGGPAIVDDNEIDAEITKFDPSGTLTNKLIDTD 689
CaurUpc2 EEYAGGAGGMHMMLDFLGGGPSIIEDHEVDKDLSMIDPEGHIAKKLIDTN 592
Consensus -XPX-XXXXXXXXXXXXXXXXX---------------------------
AfumRttA -IPP-IWAETITWIRQAVDLNS--------------------------- 380
NcraNcSR TIPPEVWGA--DWYAEEQALKDQSEHSYYRHIELLCQTGNSTRSIPEVP 517
ScerUpc2 -LPS-MTTTNF------SDFSL--------------------------- 913
ScerEcm22 -LPS-MTTTNF------SAFM---------------------------- 814
NglaUpc2A -LPS-MTTTNL------SDFM---------------------------- 922
NglaUpc2B -LPS-LVAANI------NEYL---------------------------- 844
CalbUpc2 NLPS-VLTSNLDLMQGDNGFMNMK------------------------- 712
CaurUpc2 NLPS-DITSNLDIMQGDNGFIGSNDI----------------------- 617

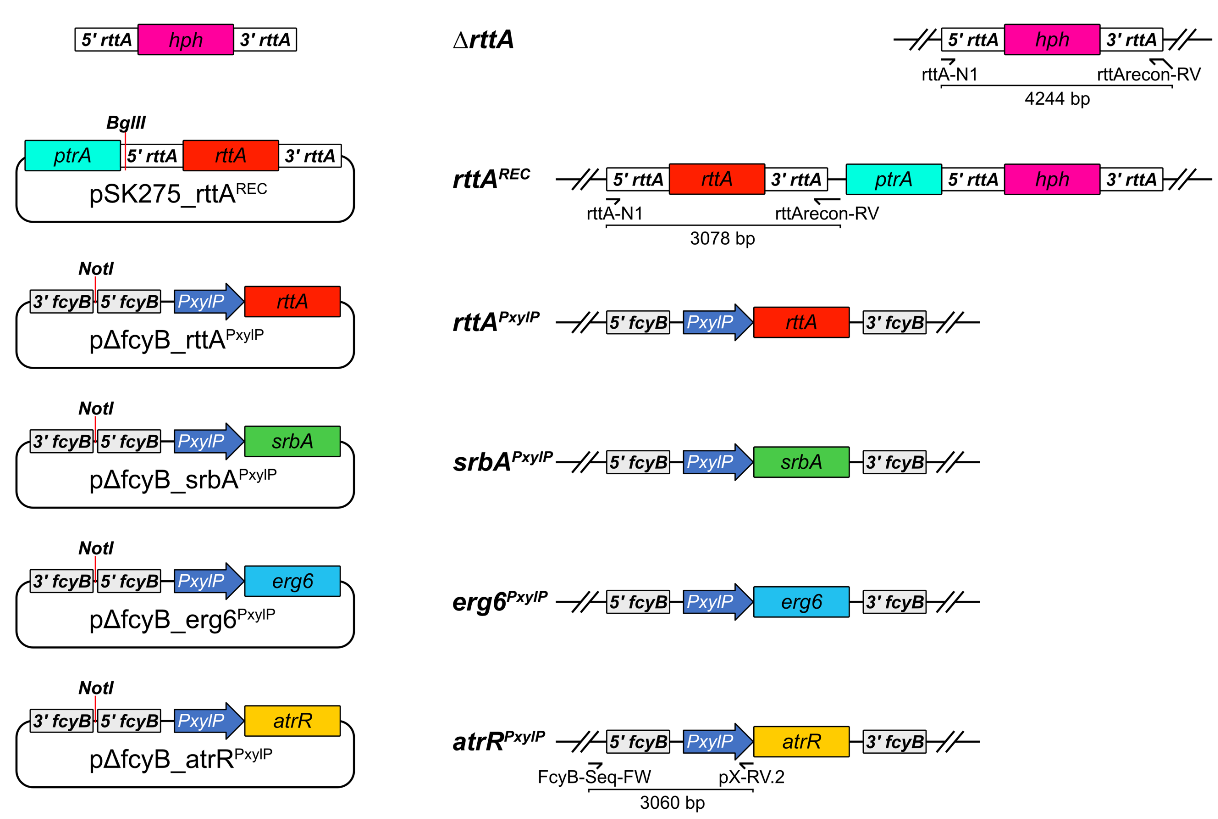

Fig S2 **Generation of *A. fumigatus* mutants.** The fusion PCR product and the plasmids used to generate the mutants are shown on the left. The corresponding resulting *A. fumigatus* genotypes are shown on the right.

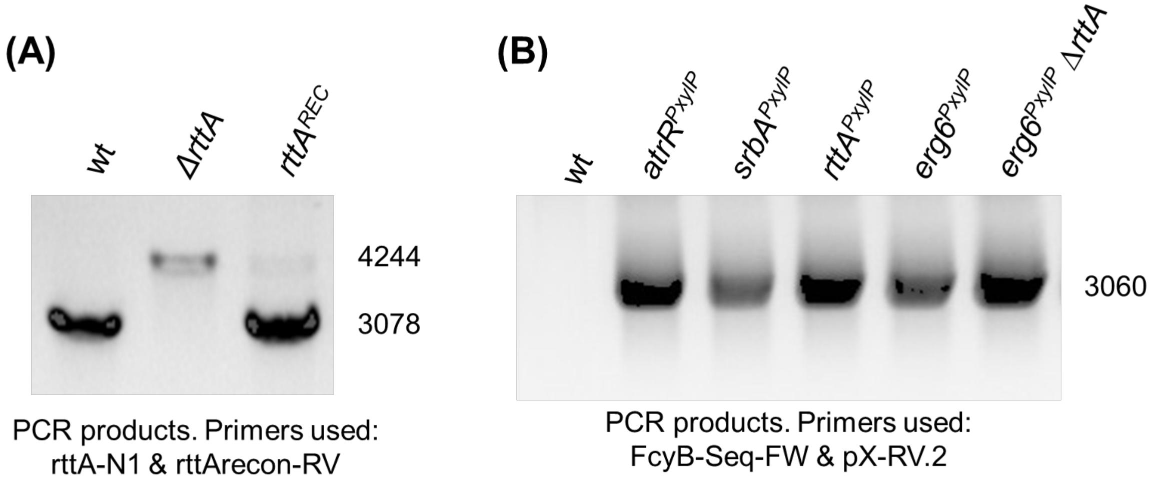

Fig S3 **PCR validation of overexpression, deletion and reconstitution mutants.** (A) The deletion and reconstitution of the *rttA* gene in the *A. fumigatus* genome were confirmed by PCR amplification of a region spanning the 5′ and 3′ flanks of the *rttA* gene. The Δ*rttA* mutant yields a larger PCR product than the wild type because the *rttA* gene has been replaced by the longer hygromycin B resistance cassette. (B) The integration of *atrR*, *srbA*, *rttA* and *erg6* overexpression cassettes into the *A. fumigatus* genome was confirmed by PCR amplification of the 5′ *fcyB* region together with the downstream *PxylP*, yielding a 3060 bp product that was detected on an agarose gel. Schematics in Fig S2 highlight the location of primers and PCR fragments that confirm correct strains.

Table S1 **DNA sequences of the previously predicted (*rttA*^{OLD}) and corrected *rttA*.** The proteins RttA^{OLD} and RttA are 309 and 380 amino acids in size, respectively. Exons are given in upper case letters, introns in lower case letters and colored in magenta.

rttA^{OLD}
ATGATTGACACAAAGCCTCAAGAGTGGCCCATGTCCACCAGAATGCTCGAGATGAGGTTGA<br>TGCATCATTATCTGACAAAAACTTACCACACCTTGCACCAAGGCAAAATCGATGCGACTCA<br>TTTCCAGACGGTCGTCCCGGAAATGGCCACATCCCACCCTTTCTTGTTGGATAGCTTACTG<br>GCACTGACAGCTCTGCACCTAGCCTTCCTGAACCCGGCCGACAAGCGCCCGTGGATGGAAG<br>CTGCGCTCAAATATCAAAATCAGGCATGTTCGGTTTTTAGTCGGGTTCTCGTGGACATCTC<br>CCCAGAGAATTGTGGACCTGCGTTTATCTGCGCGGTTTTCATCCTCCTATGTGCGACTGCA<br>TATCCATGCGTCGCCGGGGATACTCATCCGTTTGATCCACTAGCCCAGGTCCTGGAAACTC<br>GTCGTCTCCTTGTCGGGTGTGCTTTTCTTTACCATCATCTGCATCGTCATCCAGGGCAATT<br>GAAAGAGTGGCTACGGTTCCCGAACGATGTGAAACGGGAGGAAAATGTCACATCTCAAGGg<br>tgatttatcattcatttctttttgcctagtatgatagctcattctgaccgcaggacgtgga<br>atacgaagcttgtgccgctgcgcaggtacgtatattcctctgcctgcatcgttgaatggaa<br>cggctgacatcgtgcaagTGCTCTTCTAGATTCTCTACGACAAGTAGCGGCCGTCATGAAC<br>GATGCCAGTGAGCCGCACCGGGCAGCCTACCAGGAGACCTGGGATTTTCTACACGACACCA<br>TCAGTTTATGGCCATTGGGAGGTCCTCGTGGAGGCATCATATCCTGGCCCGTCCATATCGG<br>CGAAGACTACATTGCTCTGTTGAAGCAGGGAGACTGGATCGCTCGTATTTTATTTCTACAC<br>TATGGAGTTGGCATGCACCTGCTGTCGGATAAATGGTACGTTAGCGATTGGGGGCGTCGCT<br>TGGTGGCTGCGGTCTTGCAGCCTCTGCAGGACATTCCTCCAATCTGGGCCGAAACCATCAC<br>TTGGATACGACAGGCAGTCGATCTCAACAGCTAG
rttA (corrected)
ATGCCACCGCGTCGCAGTCATACTAAATCCCGGAAGGGATGTACTAACTGCAAGAAGCGCC<br>ATGTCAAATGCGATGAAGAGCTACCTAGgtgcgactgctccaatttatcggccgtcccttc<br>gcaccaacgggctttgaccattcagcgccatgctgtcaaggctacatgtcaatcattctgt<br>gaaccaaatactgactcgattacagTTGTGGGTTATGCAAGAAACGGCAGCTAGAATGCAT<br>CTATCAATCATCGGCCAGTGGAGTCGACAGCCAACGTGCATCTACCCCGCGAGAGATGATT<br>GACACAAAGCCTCAAGAGTGGCCCATGTCCACCAGAATGCTCGAGATGAGGTTGATGCATC<br>ATTATCTGACAAAAACTTACCACACCTTGCACCAAGGCAAAATCGATGCGACTCATTTCCA<br>GACGGTCGTCCCGGAAATGGCCACATCCCACCCTTTCTTGTTGGATAGCTTACTGGCACTG<br>ACAGCTCTGCACCTAGCCTTCCTGAACCCGGCCGACAAGCGCCCGTGGATGGAAGCTGCGC<br>TCAAATATCAAAATCAGGCATGTTCGGTTTTTAGTCGGGTTCTCGTGGACATCTCCCCAGA<br>GAATTGTGGACCTGCGTTTATCTGCGCGGTTTTCATCCTCCTATGTGCGACTGCATATCCA<br>TGCGTCGCCGGGGATACTCATCCGTTTGATCCACTAGCCCAGGTCCTGGAAACTCGTCGTC<br>TCCTTGTCGGGTGTGCTTTTCTTTACCATCATCTGCATCGTCATCCAGGGCAATTGAAAGA<br>GTGGCTACGGTTCCCGAACGATGTGAAACGGGAGGAAAATGTCACATCTCAAGGgtgattt<br>atcattcatttctttttgcctagtatgatagctcattctgaccgcagGACGTGGAATACGA<br>AGCTTGTGCCGCTGCGCAGgtacgtatattcctctgcctgcatcgttgaatggaacggctg<br>acatcgtgcaagTGCTCTTCTAGATTCTCTACGACAAGTAGCGGCCGTCATGAACGATGCC<br>AGTGAGCCGCACCGGGCAGCCTACCAGGAGACCTGGGATTTTCTACACGACACCATCAGTT<br>TATGGCCATTGGGAGGTCCTCGTGGAGGCATCATATCCTGGCCCGTCCATATCGGCGAAGA<br>CTACATTGCTCTGTTGAAGCAGGGAGACTGGATCGCTCGTATTTTATTTCTACACTATGGA<br>GTTGGCATGCACCTGCTGTCGGATAAATGGTACGTTAGCGATTGGGGGCGTCGCTTGGTGG<br>CTGCGGTCTTGCAGCCTCTGCAGGACATTCCTCCAATCTGGGCCGAAACCATCACTTGGAT<br>ACGACAGGCAGTCGATCTCAACAGCTAG
RttA^{OLD} (309 amino acids)
MIDTKPQEWPMSTRMLEMRLMHHYLTKTYHTLHQGKIDATHFQTVVPEMATSHPFLLDSLL<br>ALTALHLAFLNPADKRPWMEAALKYQNQACSVFSRVLVDISPENCGPAFICAVFILLCATA<br>YPCVAGDTHPFDPLAQVLETRRLLVGCAFLYHHLHRHPGQLKEWLRFPNDVKREENVTSQG

ALLDSLRQVAAVMNDASEPHRAAYQETWDFLHDTISLWPLGGPRGGIISWPVHIGEDYIAL LKQGDWIARILFLHYGVGMHLLSDKWYVSDWGRRLVAAVLQPLQDIPPIWAETITWIRQAV DLNS
RttA (380 amino acids)
MPPRRSHTKSRKGCTNCKKRHVKCDEELPSCGLCKKRQLECIYQSSASGVDSQRASTPREM IDTKPQEWPMSTRMLEMRLMHHYLTKTYHTLHQGKIDATHFQTVVPEMATSHPFLLDSLLA LTALHLAFLNPADKRPWMEAALKYQNQACSVFSRVLVDISPENCGPAFICAVFILLCATAY PCVAGDTHPFDPLAQVLETRRLLVGCAFLYHHLHRHPGQLKEWLRFPNDVKREENVTSQGT WNTKLVPLRSALLDSLRQVAAVMNDASEPHRAAYQETWDFLHDTISLWPLGGPRGGIISWP VHIGEDYIALLKQGDWIARILFLHYGVGMHLLSDKWYVSDWGRRLVAAVLQPLQDIPPIWA ETITWIRQAVDLNS

Table S4 **The strains used in this study.** A1160P+ (1) served as the basis for all mutants generated and used in this study.

Strain	Genotype	Reference
A1160P+ (wt)	Δ*ku80::pyrG*	(1)
Δ*atrR*	Δ*atrR::hph*	(2)
Δ*srbA*	Δ*srbA::hph*	(3)
Δ*rttA*	Δ*rttA::hph*	This study
$atrR^{PxylP}$	Δ*fcyB::PxylP-atrR*	This study
$srbA^{PxylP}$	Δ*fcyB::PxylP-srbA*	This study
$rttA^{PxylP}$	Δ*fcyB::PxylP-rttA*	This study
$erg6^{PxylP}$	Δ*fcyB::PxylP-erg6*	This study
$rttA^{REC}$	Δ*rttA::hph; rttA, ptrA*	This study
$erg6^{PxylP}\Delta rttA$	Δ*fcyB::PxylP-erg6*Δ*rttA::hph*	This study

References

1. Fraczek MG, Bromley M, Buied A, Moore CB, Rajendran R, Rautemaa R, Ramage G, Denning DW, Bowyer P. 2013. The *cdr1B* efflux transporter is associated with non-*cyp51a*-mediated itraconazole resistance in *Aspergillus fumigatus*. Journal of Antimicrobial Chemotherapy 68:1486-1496.
2. Furukawa T, van Rhijn N, Fraczek M, Gsaller F, Davies E, Carr P, Gago S, Fortune-Grant R, Rahman S, Gilsenan JM, Houlder E, Kowalski CH, Raj S, Paul S, Cook P, Parker JE, Kelly S, Cramer RA, Latge JP, Moye-Rowley S, Bignell E, Bowyer P, Bromley MJ. 2020. The negative cofactor 2 complex is a key regulator of drug resistance in *Aspergillus fumigatus*. Nature Communications 11.
3. Gsaller F, Hortschansky P, Furukawa T, Carr PD, Rash B, Capilla J, Muller C, Bracher F, Bowyer P, Haas H, Brakhage AA, Bromley MJ. 2016. Sterol Biosynthesis and Azole Tolerance Is Governed by the Opposing Actions of SrbA and the CCAAT Binding Complex. Plos Pathogens 12.

Table S5 **The primers used in this study.**

Primer name	Sequence (5′ → 3′)
**Generation and validation of strains**	
hph-FW	CCGGCTCGGTAACAGAACTAACGGCGTAACCAAAAGTCAC
hph-RV	GGGAGCATATCGTTCAGAGCTCTTGACGACCGTTGATCTG
rttA-1	TGCTTCTAGCACCCTTTTCG
rttA-2	TAGTTCTGTTACCGAGCCGGTAGTACGCCAAGGAGGGATG
rttA-3	GCTCTGAACGATATGCTCCCCGCGAGGCTTTCTGAATTAC
rttA-4	CTCACAGCATCAGCAGCATT
rttA-N1	CGCGAAGAGCATCATTATCA
rttA-N2	TGATTTCCACCATTGGGATT
pX-FW.2	CCATGGCAGCAGTGATTTCA
pX-RV.2	GGTTGGTTCTTCGAGTCGATG
atrR-FW	ATCGACTCGAAGAACCAACCATGGAGTCTTTACTGCGTCTT
atrR-RV	GAAATCACTGCTGCCATGGTCACAATCCACCGTGGGG
srbA-FW	ATCGACTCGAAGAACCAACCATGTCCACCCCCGGCATT
srbA-RV	GAAATCACTGCTGCCATGGTCACGACTCATCGGAGAGCAGT
rttA-FW	ATCGACTCGAAGAACCAACCATGCCACCGCGTCGCAGT
rttA-RV	GAAATCACTGCTGCCATGGCTAGCTGTTGAGATCGACTGCCT
erg6-FW	ATCGACTCGAAGAACCAACCATGGCCCCCGTAGCTTTG
erg6-RV	TGAAATCACTGCTGCCATGGTTACTCGGGCTTGCGTCC
BBpSK275-FW	GCTTATCGATACCGTCGACCT
BBpSK275-RV	AATGCCCCACCGTTACATAC
rttArecon-FW	GTATGTAACGGTGGGGCATTAACATCTATGATGCTTCATGAGAATCATGA
rttArecon-RV	GGTCGACGGTATCGATAAGCGTTTCTCCACATCTTGGAGTGTCC
FcyB-Seq-FW	AGCTCGACTCAGGGCAATTA
**qPCR analysis**	
RT-gpdA-FW	GAGCTCAAAAACATCCTCGGC
RT-gpdA-RV	CGAAGTTGGGGTTGAGGGAG
RT-SrbA-FW	CGTGGACATTCAACGTATCG
RT-SrbA-RV	ACAAGGTGGCCAAATGACTC
RT-AtrR-FW	AATACTATGGCCGACGATGC
RT-AtrR-RV	CGGGCATAGCCTGTATGTTT
RT-rttA-FW	ATCATCTGCATCGTCATCCA
RT-rttA-RV	GGCCGCTACTTGTCGTAGAG
RT-erg6-FW	TCTGCGGAGCTATGGAAACT
RT-erg6-RV	AATACATGGGCGTGAAGAGC
RT-Cyp51A-FW	AAGGAGCAGGAGAACGACAA
RT-Cyp51A-RV	GCCAGAATCACACCAAGGTT
RT-Cyp51B-FW	CACCATCAGTTATGGCATCG
RT-Cyp51B-RV	GCTTGCCGTTTAGGATGAAG
RT-abcA-FW	TAGGAATAGGGTCCGCATTG
RT-abcA-RV	CATGAGTTTCTCCCGCAATC
RT-MFS-FW	CTCAGATTGCCAATGTCTGC
RT-MFS-RV	CAGCTTTGGTAGGCGTGTCT
RT-Cdr1B-FW	TTACCAAGCCTCCCAGAGTG
RT-Cdr1B-RV	ACGTTCCGGACATTCAAATC
